# Altered macronutrient composition and genetics influence the complex transcriptional network associated with adiposity in the Collaborative Cross

**DOI:** 10.1186/s12263-022-00714-x

**Published:** 2022-08-10

**Authors:** Phoebe Yam, Melissa VerHague, Jody Albright, Erik Gertz, Fernando Pardo-Manuel de Villena, Brian J. Bennett

**Affiliations:** 1 Integrative Genetics and Genomics Graduate Group, University of California Davis, Davis, CA USA; 2 Agricultural Research Service, US Department of Agriculture, Western Human Nutrition Research Center, Davis, CA USA; 3 Nutrition Research Institute, University of North Carolina Kannapolis, Kannapolis, NC USA; 4 Department of Genetics, Lineberger Comprehensive Cancer Center, University of North Carolina, Chapel Hill, Chapel Hill, NC USA; 5 Department of Nutrition, University of California Davis, Davis, CA USA

**Keywords:** Collaborative Cross, Diet, Nutrigenomics, Genetics, Obesity, WGCNA, Hepatic gene expression

## Abstract

**Background:**

Obesity is a serious disease with a complex etiology characterized by overaccumulation of adiposity resulting in detrimental health outcomes. Given the liver’s critical role in the biological processes that attenuate adiposity accumulation, elucidating the influence of genetics and dietary patterns on hepatic gene expression is fundamental for improving methods of obesity prevention and treatment. To determine how genetics and diet impact obesity development, mice from 22 strains of the genetically diverse recombinant inbred Collaborative Cross (CC) mouse panel were challenged to either a high-protein or high-fat high-sucrose diet, followed by extensive phenotyping and analysis of hepatic gene expression.

**Results:**

Over 1000 genes differentially expressed by perturbed dietary macronutrient composition were enriched for biological processes related to metabolic pathways. Additionally, over 9000 genes were differentially expressed by strain and enriched for biological process involved in cell adhesion and signaling. Weighted gene co-expression network analysis identified multiple gene clusters (modules) associated with body fat % whose average expression levels were influenced by both dietary macronutrient composition and genetics. Each module was enriched for distinct types of biological functions.

**Conclusions:**

Genetic background affected hepatic gene expression in the CC overall, but diet macronutrient differences also altered expression of a specific subset of genes. Changes in macronutrient composition altered gene expression related to metabolic processes, while genetic background heavily influenced a broad range of cellular functions and processes irrespective of adiposity. Understanding the individual role of macronutrient composition, genetics, and their interaction is critical to developing therapeutic strategies and policy recommendations for precision nutrition.

**Supplementary Information:**

The online version contains supplementary material available at 10.1186/s12263-022-00714-x.

## Background

Obesity is characterized by the disproportionate and excessive accumulation of adipose tissue relative to an individual’s height, resulting in decreased health and increased risk of developing a myriad of chronic diseases such as atherosclerosis, cardiovascular disease, metabolic syndrome, type 2 diabetes, and certain types of cancer [97]. The simplest definition of obesity is excessive adiposity resulting from the chronic imbalance between energy intake and expenditure. The underlying mechanisms involved in maintaining energy balance are complex and regulated by numerous factors such as genetic background [3, 52, 82], metabolism [19, 84, 90], gut microbiome [36, 57, 91], and environmental factors such as diet in the context of overfeeding [11–13, 76, 81]. Additionally, the specific interaction of dietary macronutrients and the endocrine system, in particular insulin response and signaling, has a critical role in the etiology of obesity [53]. Differences in dietary macronutrient composition can influence substrate utilization; specifically, rapidly digestible carbohydrates may interact with insulin and other hormones to increase fat accumulation relative to other macronutrients.

In addition to the complex interactions between adipose tissue, the central nervous system, nutrients, and hormones that regulate energy balance [3, 25], the liver also influences the susceptibility to obesity, given its major role in the metabolism and processing of macronutrients including glycogenolysis, production of triglycerides, lipogenesis, and the synthesis of amino acids, cholesterol, and lipoproteins [75, 93]. Obesity in turn can induce the pathological response of insulin resistance in the liver, which results in an impaired ability of insulin to decrease glucose output from the liver while continuing to stimulate lipogenesis; this disruption of appropriate carbohydrate and lipid metabolism is thought to contribute to some of the health complications associated with obesity like metabolic syndrome and cardiovascular disease. Adipokines such as adiponectin, adipocyte dysfunction, metabolism, and circulating metabolite levels affect hepatic gene expression [21, 56], which regulates the mechanisms involved in lipid processing, determination of metabolic rate, and other physiological processes associated with energy imbalance [46, 93]. Furthermore, an individual’s inherent genetic architecture and specific environmental exposures such as diet also shape hepatic gene expression [31, 41, 80]. Given that the liver regulates so many biological processes related to obesity development, elucidating the effects of genetic architecture and diet on hepatic gene expression is necessary to understand the mechanisms underlying susceptibility to obesity and development of effective prevention and treatment regimes.

Modern molecular biology techniques have revolutionized our ability to detect changes in gene expression [50, 74], which allows one to infer potential candidate genes and pathways underlying metabolic dysfunction [16, 33]. Identification of genes and pathways that determine susceptibility to obesity facilitates the understanding of the underlying mechanisms behind the development of obesity, which is instrumental to determining effective methods of prevention and treatment. Simultaneous to the advances in high-throughput assessment of gene expression, a novel population of mice has been developed. Derived from elaborate intercrosses of eight founder mouse strains [7, 35, 89], the CC is a large recombinant inbred mouse population with tremendous genetic diversity and genetic contribution from five classically inbred strains, A/J, C57BL/6J (B6), 129S1/SvImJ (129), NOD/ShiLtJ (NOD), and NZO/HILtJ (NZO), and three wild-derived strains, CAST/EiJ (CAST), PWK/PhJ (PWK), and WSB/EiJ (WSB) [9, 64, 79, 85]. The genetic and phenotypic diversity of the CC is of similar scale to the human population [86] and provides an opportunity to address the complex interactions between genetics and dietary macronutrient composition that affect hepatic gene expression. The ability to utilize multiple replicates of individual CC strains allows for more precise delineation between confounding environmental influences and dietary effects within the context of a known genetic architecture.

Previously, we examined the effects of diet and genetic background on adiposity and other obesity-related traits [101]. In the current study, we focus on the effects of macronutrient composition and strain (genetic background) on hepatic gene expression and relate these to phenotypic traits and biological functions. To find potential candidate genes or functional pathways underlying metabolic dysfunction regulated by diet in a genetically diverse population, we administered a challenge of either high-protein (HP) or high-fat high-sucrose (HS) diet to 22 strains of mice from the Collaborative Cross (CC) mouse panel for 8 weeks and performed microarray gene expression analysis of 11,542 genes using high-quality RNA from liver tissue, in addition to extensive phenotyping. To ascertain the expression of genes (mRNA) associated with adiposity, determine which genes were differentially expressed by dietary macronutrients and genetic strain, and identify groups of related genes affected by genetic background and/or diet in the liver, we examined hepatic gene expression levels and related them to phenotypes using one analyses pipeline centered around linear models for microarray (limma) and a separate analyses pipeline focused on weighted gene co-expression network analysis (WGCNA) (see Supplementary Fig. 1, Additional file 1), which facilitated exploration of gene expression from two perspectives: for individual genes using the limma approach and for groups of genes using the network approach.

## Results

### Diet-induced adiposity was correlated with the expression level of thousands of genes in the liver

**Fig. 1:**
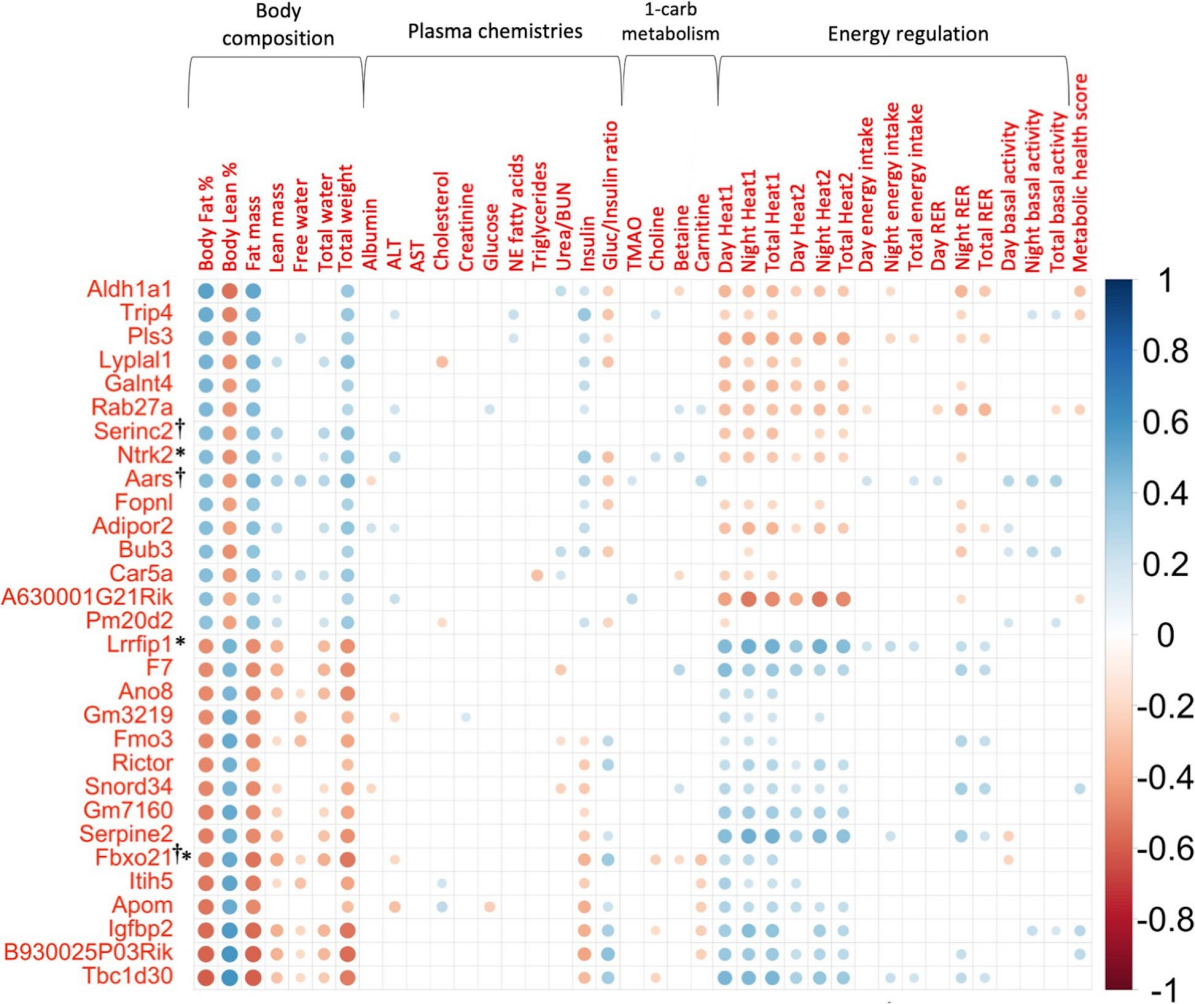
Top 30 genes with expression levels most significantly correlated with body fat %. Multiple biweight midcorrelations (bicor) and their corresponding Student correlation *p*-values were calculated between phenotypic data and microarray liver gene expression data to properly take into account the actual number of observations when determining which genes’ expression levels were correlated with post-diet phenotypes of interest. The top 15 genes whose expression is most significantly positively correlated with body fat % (bicor ≥ 0.410, p ≤ 2.53 × 10^−6^) and top 15 genes whose expression is most significantly negatively correlated with body fat % (bicor ≤ −0.466, *p* ≤ 5.42 × 10^−8^) are shown. With the exception of insulin and glucose/insulin ratio, most of the top 30 genes’ expression most significantly correlated with body fat % were not significantly correlated with circulating analytes but were significantly correlated with metabolic (energy regulation) traits. Genes are ordered on the *y*-axis in descending order of bicor with the strongest positive correlation at the top and the strongest negative correlation at the bottom. Scale indicates bicor value with color darkness as indicator of correlation strength. †Indicates genes that are also differentially expressed by diet; all 30 genes were found to be differentially expressed by strain. *Indicates genes found to be associated with at least one obesity-related trait in humans according to the GWAS catalog. Annotation for all genes with expression significantly correlated with body fat % are shown in Supplementary Table 1, Additional file 2. All significant correlations in this figure are shown in Supplementary Table 2, Additional file 2

Microarray gene expression analysis of 11,542 genes was performed using high-quality RNA from livers of 123 CC mice collected after an 8-week challenge of either a high-protein (HP) or high-fat high-sucrose (HS) diet. Correlations of post-diet adiposity with normalized gene expression levels using calculations of multiple biweight midcorrelations (bicor) and their corresponding Student correlation *p*-values were performed to determine which genes’ expression levels were associated with body fat % and relevant traits. Post-diet body fat % was significantly correlated with the expression of 2552 genes out of 11,542 genes expressed in the liver with validated annotation (Supplementary Table 1, Additional file 2), with the top 15 most significant positive and 15 most significant negative correlations shown in Fig. 1 (Supplementary Table 2, Additional file 2). Specifically, post-diet body fat % showed significant moderate negative correlation with the gene expression of TBC1 domain family (*Tbc1d30*; bicor = −0.603, *p* = 1.56 × 10^−13^), insulin-like growth factor binding protein 2 (*Igfbp2*; bicor = −0.560, *p* = 1.62 × 10^−11^), apolipoprotein M (*ApoM*; bicor = −0.530, *p* = 2.82 × 10^−10^), inter-alpha globulin inhibitor H5 (*Itih5*; bicor = −0.527, *p* = 3.76 × 10^−10^), and flavin containing monooxygenase 3 (*Fmo3*; bicor = −0.484, *p* = 1.44 × 10^−8^), as well as moderate positive correlation between post-diet adiposity and gene expression of aldehyde dehydrogenase (*Aldh1a1*; bicor = 0.539, *p* = 1.29 × 10^−10^), thyroid hormone receptor interactor 4 (*Trip4*; bicor = 0.494, *p* = 6.41 × 10^−9^), plastin 3 (*Pls3*; bicor = 0.470, *p* = 4.17 × 10^−8^), lysophospholipase-like 1 (*Lyplal1*; bicor = 0.468, *p* = 4.81 × 10^−8^), and adiponectin receptor 2 (*Adipor2*; bicor = 0.426, *p* = 9.21 × 10^−7^). Of these highly correlated genes, metabolic health score was also significantly correlated with *Aldh1a1* (bicor = −0.282, *p* = 1.63 × 10^−3^), *Trip4* (bicor = −0.246, *p* = 6.24 × 10^−3^), and *Igfbp2* (bicor = 0.270, *p* = 2.61 × 10^−3^); total weight was also significantly mildly correlated with the expression levels of these top 30 genes (Fig. 1).

The expression levels of many genes that were significantly correlated either negatively or positively with body fat % were also reciprocally correlated with lean % and heat production (Fig. 1). Very few of the expression levels of the top 30 genes showed significant correlations with circulating analytes or metabolites except insulin and glucose/insulin ratio. Accordingly, body fat % was also significantly correlated both positively and negatively with expression levels of genes encoding proteins instrumental to insulin signaling that regulate metabolic pathways [2, 14, 17, 26, 30, 42, 43, 59, 104], including insulin-degrading enzyme (*Ide*; bicor = 0.348, *p* = 7.85 × 10^−5^), phosphoinositide-3-kinase regulatory subunit 1 (*Pik3r1*; bicor = 0.211, *p* = 0.019), insulin-induced gene 1 (*Insig1*; bicor = 0.196, *p* = 0.029), insulin receptor substrate 2 (*Irs2*; bicor = −0.309, *p* = 4.98 × 10^−4^), insulin receptor (*Insr*; bicor = −0.242, *p* = 6.94 × 10^−3^), and Janus kinase 1 (*Jak1*; bicor = −0.201, *p* = 0.026).

### Differential gene expression analysis identified 1344 genes responsive to differences in dietary macronutrient composition

**Fig. 2:**
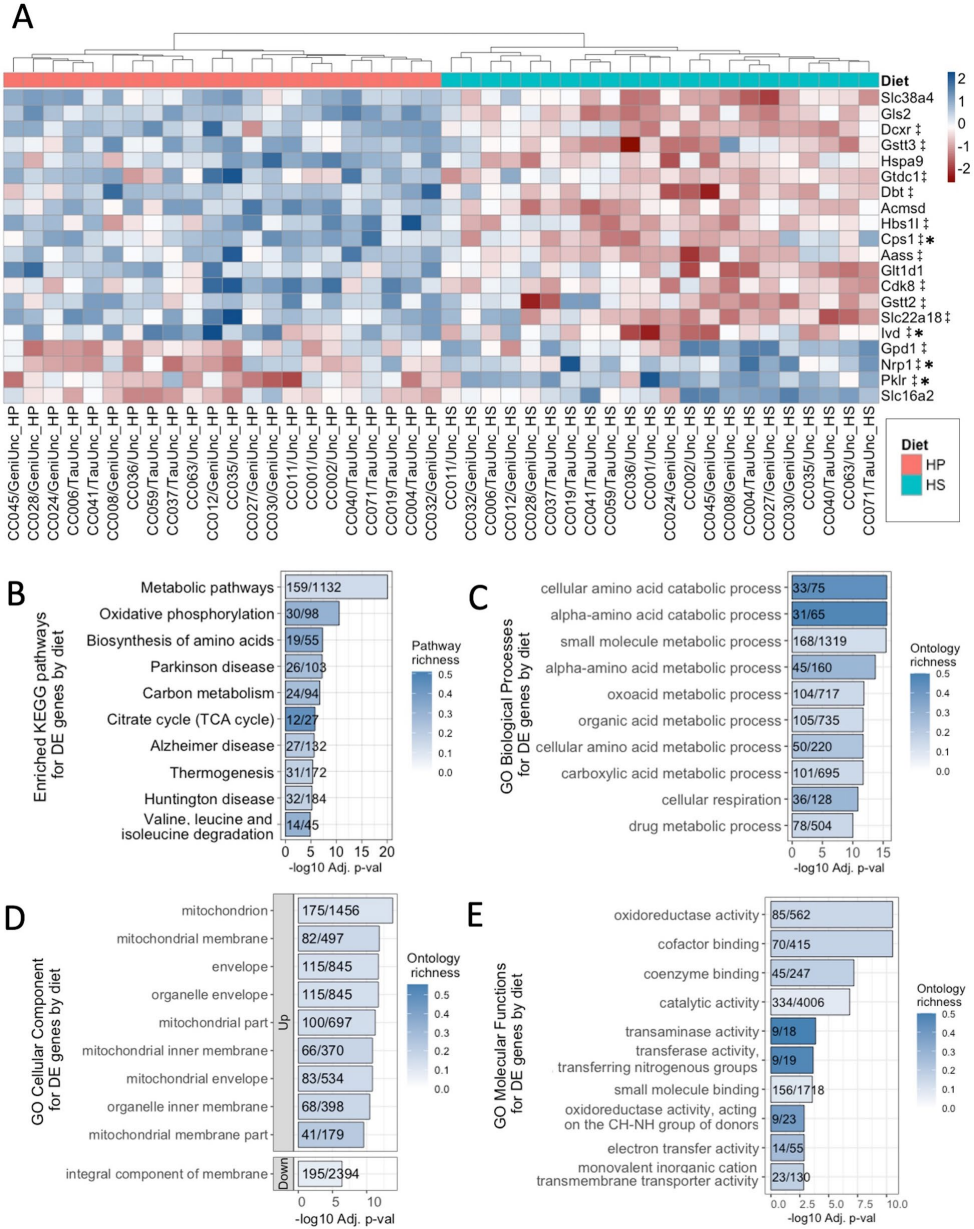
Expression patterns and enrichment of diet DEGs. **A** The top 20 most significant (BH-adjusted *p* ≤ 2.37 × 10^−8^) diet DE genes’ average Z scores of median robust multi-array average (RMA) normalized gene expression for each CC strain on either the high-protein (HP) or high-fat high-sucrose (HS) diet shown ordered from top to bottom by level of gene expression on the HP diet (highest to lowest). The genes’ average Z scores for each CC strain and diet are clustered by Euclidean distance on the *x*-axis. ‡Denotes genes also differentially expressed by strain. *Indicates genes with human homologs found in the GWAS catalog to be associated with at least one obesity-related trait. Annotation and limma results are shown for all diet DEGs in Supplementary Table 3, Additional file 2. Limma analysis of microarray data revealed genes differentially expressed by diet showing significant enrichment (*p* adj < 0.05) for **B** KEGG (20 total), **C** GO biological pathways (105 total), **D** GO cellular components (45 total), and **E** GO molecular functions (37 total). Pathways are ordered from top to bottom by significance (highest to lowest) and colored by gene richness. The top 10 enrichments for each ontology category were all upregulated on the HP diet, except for the GO cellular component and “integral component of membrane,” which was downregulated. All significant enrichment terms and enrichment analysis results are shown in Supplementary Table 4, Additional file 2

Both genetics and environmental factors such as diet are critical determinants of obesity. Although genetics have a stronger effect on susceptibility to developing obesity than diet alone [10, 29], the role of diet as an environmental factor that influences gene expression is still important, since changes in dietary patterns can help mitigate the degree of obesity that develops by altering gene expression levels. To assess which genes’ expression levels are affected by diet, differential gene expression analysis was performed using the R package limma (linear models for microarray) on liver gene expression data. Comparing the HS diet to the HP diet revealed 1344 genes that were differentially expressed by diet (*p* adj < 0.05, Supplementary Table 3, Additional file 2) with the top 20 most significant hits showing patterns of expression clustering by diet (Fig. 2A), where 16 genes showed increased expression and 4 genes showing decreased expression in mice fed the HP diet relative to the HS diet, though expression patterns exhibited some degree of inter-strain variation depending on the gene and strain. The opposite patterns of expression for these genes were shown in mice fed the HS diet, i.e., genes that showed increased expression in mice fed the HP diet had decreased levels of expression in mice fed the HS diet (Fig. 2A). The expression levels of 389 differentially expressed genes (DEGs) by diet were significantly correlated with body fat % (*p* < 0.05), including *Irs2* and *Pik3r1*.

The Kyoto Encyclopedia of Genes and Genomes (KEGG) pathway and gene ontology (GO) enrichment analyses identified 20 significantly overrepresented KEGG pathways and 187 significantly overrepresented GO terms for DEGs by diet (Fig. 2B–E; see Supplementary Table 4, Additional file 2), with varying degrees of gene richness defined by the number of up- or downregulated DEGs found belonging to each KEGG pathway or GO term out of the total number of genes that comprise each KEGG pathway or GO term. The most significantly overrepresented KEGG pathways identified were metabolic pathways, oxidative phosphorylation, and biosynthesis of amino acids (*p* adj ≤ 5.05 × 10^−8^). In terms of each GO term category, 105 GO biological processes, 45 GO cellular components, and 37 GO molecular functions were significantly overrepresented (*p* adj < 0.05), with the top 10 most significantly overrepresented GO terms in DEGs by diet shown in Fig. 2C–E. The majority of enrichment terms were related to metabolism of a wide variety of substrates with numerous enrichments of mitochondrial cellular components (see Supplementary Table 4, Additional file 2).

### Genetic architecture perturbed global hepatic gene expression to a greater extent than macronutrient composition

**Fig. 3:**
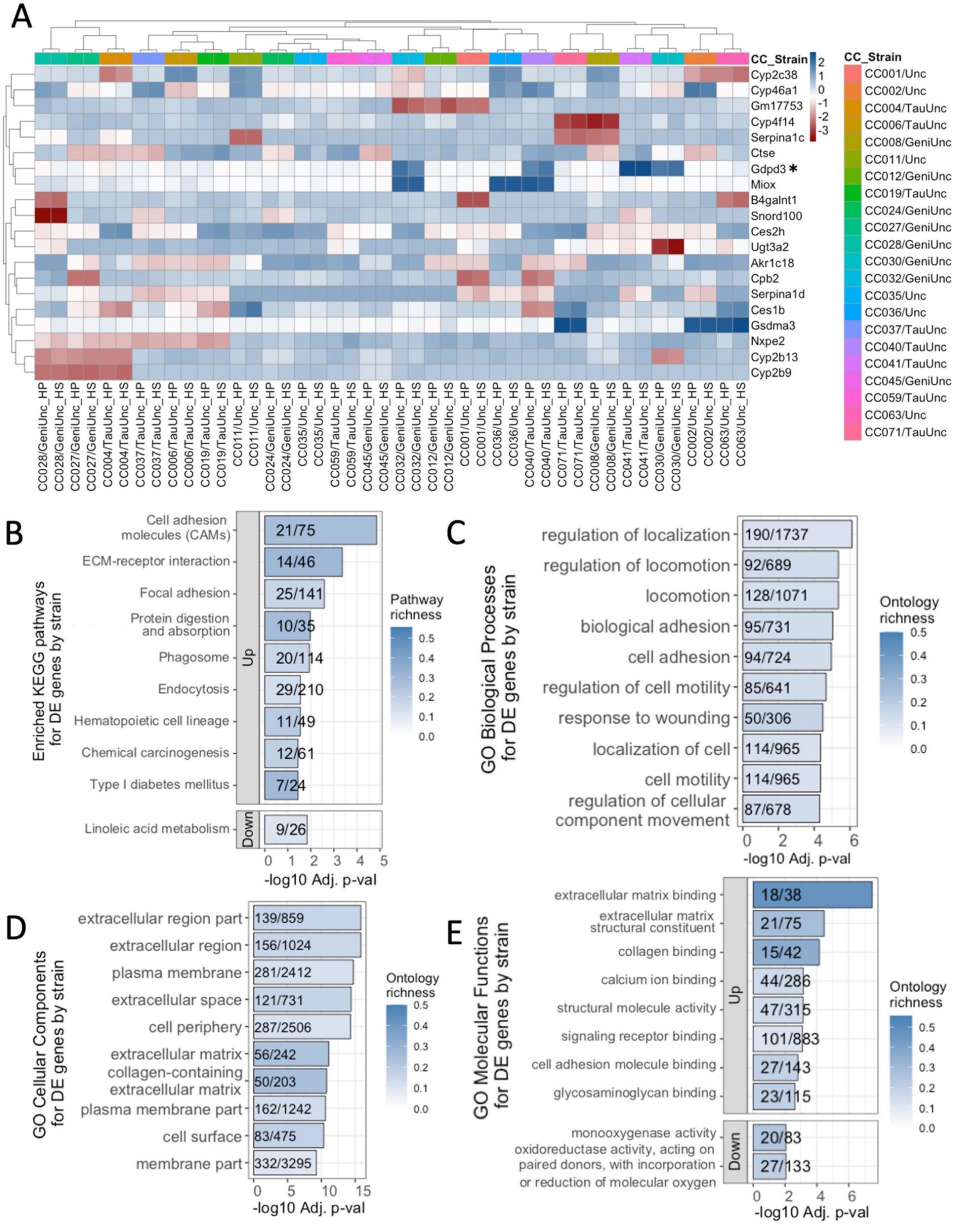
Expression patterns and enrichment of transcripts differentially expressed by CC strain. **A** The top 20 most significant (BH-adjusted *p* ≤ 2.631 × 10^−56^) strain DE genes’ average Z scores of median robust multi-array average (RMA) normalized, gene expression for each CC strain on either the high-protein (HP) or high-fat high-sucrose (HS) diet shown. Gene average RMA Z scores for each CC strain and diet are clustered according to Euclidean distance by CC strain and diet on the *x*-axis and by gene on the *y*-axis. The human homolog of *Gdpd3* was found in the GWAS catalog to be associated with at least one obesity-related trait. Annotation and limma results are shown for all strain DEGs in Supplementary Table 5, Additional file 2. Limma analysis of microarray data revealed genes differentially expressed by strain showing significant enrichment (*p* adj < 0.05) for **B** KEGG (13 total), **C** GO biological pathways (95 total), **D** GO cellular components (44 total), and **E** GO molecular functions (24 total). Pathways are ordered from top to bottom by significance (highest to lowest) and colored by gene richness. The top 10 enrichments for each ontology category were all upregulated on the HP diet, except for the linoleic acid metabolism KEGG pathway, and GO molecular functions “monooxygenase activity” and “oxidoreductase activity, acting on paired donors…,” which were downregulated. All significant enrichment terms and enrichment analysis results are shown in Supplementary Table 6, Additional file 2

Genetics is clearly an important factor affecting susceptibility to metabolic dysfunction. We tested the role of genetics in regulatory gene expression by performing limma differential gene expression analysis by CC strain. Differential gene expression analysis revealed 9436 DEGs by CC strain (*p* adj < 0.05, Supplementary Table 5, Additional file 2), with the top 20 most significant hits showing patterns of expression clustering by CC strain instead of diet (Fig. 3A). Unlike the inter-strain variation of expression patterns for diet DEGs, expression patterns were consistent across diets for strain DEGs. DEGs by CC strain showed similar levels of expression within each CC strain regardless of the diet fed. One-thousand one-hundred thirty-one DEGs by CC strain were also differentially expressed by diet (such as *Irs2* and *Pik3r1*), and 2367 of DEGs by CC strain were correlated with body fat % (nominal *p* < 0.05), including *Ide*, *Insig1*, *Irs2*, *Jak1*, and *Pik3r1*. Interestingly, additional genes encoding proteins crucial to insulin signaling [2, 5, 6, 30, 60, 100] were differentially expressed by strain but not diet, specifically high mobility group AT-hook 1 (*Hmga1*), insulin-induced gene 2 (*Insig2*), and insulin receptor substrate 1 (*Irs1*) (Supplementary Table 5, Additional file 2).

KEGG pathway and GO enrichment analyses identified fewer overrepresented KEGG pathways and GO terms for genes differentially expressed by CC strain than diet. For strain DEGs, 13 significantly overrepresented KEGG pathways and 163 significantly overrepresented GO terms were identified (*p* adj < 0.05, Fig. 3B–E; see Supplementary Table 6, Additional file 2), with varying degrees of gene richness. The most significantly overrepresented KEGG pathways identified were cell adhesion molecules (CAMs), ECM-receptor interaction, and focal adhesion (*p* adj ≤ 2.6 × 10^−3^), which are pathways important to cell signaling and structural binding between cells. For each GO term category, 95 GO biological processes, 44 GO cellular components, and 24 GO molecular functions were significantly overrepresented in strain DEGs (*p* adj < 0.05), with the top 10 most significantly overrepresented GO terms in DEGs by strain shown in Fig. 3C–E. In contrast to the enrichments for diet DEGs, very few of the enrichments for strain DEGs were related to metabolism. Instead, most enrichment terms were related to basal biological functions such as cell or tissue motility, cell division, tissue development, and substrate binding; most cellular compartment enrichments were derivatives of the cell membrane as opposed to the mitochondria (Supplementary Table 6, Additional file 2).

### A query of the GWAS catalog identified DEGs in the CC that were associated with obesity-related traits in humans

We were next interested in identifying clinically important genes that are suspected of causing underlying complex traits in humans to provide context for our findings relative to human obesity. Using the GWAS catalog to guide our search, we found that 15.8% of the genes expressed in the liver in this study (1819/11,542) have been previously found to be associated with obesity traits in humans [4]. Of these 1819 genes expressed in the livers of the CC mice that were also found associated with obesity traits in humans, greater than 85% (1570/1819) were found to be diet DEGs, strain DEGs, or significantly correlated with body fat % in this CC study. Using the CC as a model for obesity, we identified over 1500 genes expressed in the liver whose expression levels were either under genetic regulation, influenced by diet, or correlated with body fat %, which were also clinically important in humans.

**Fig. 4:**
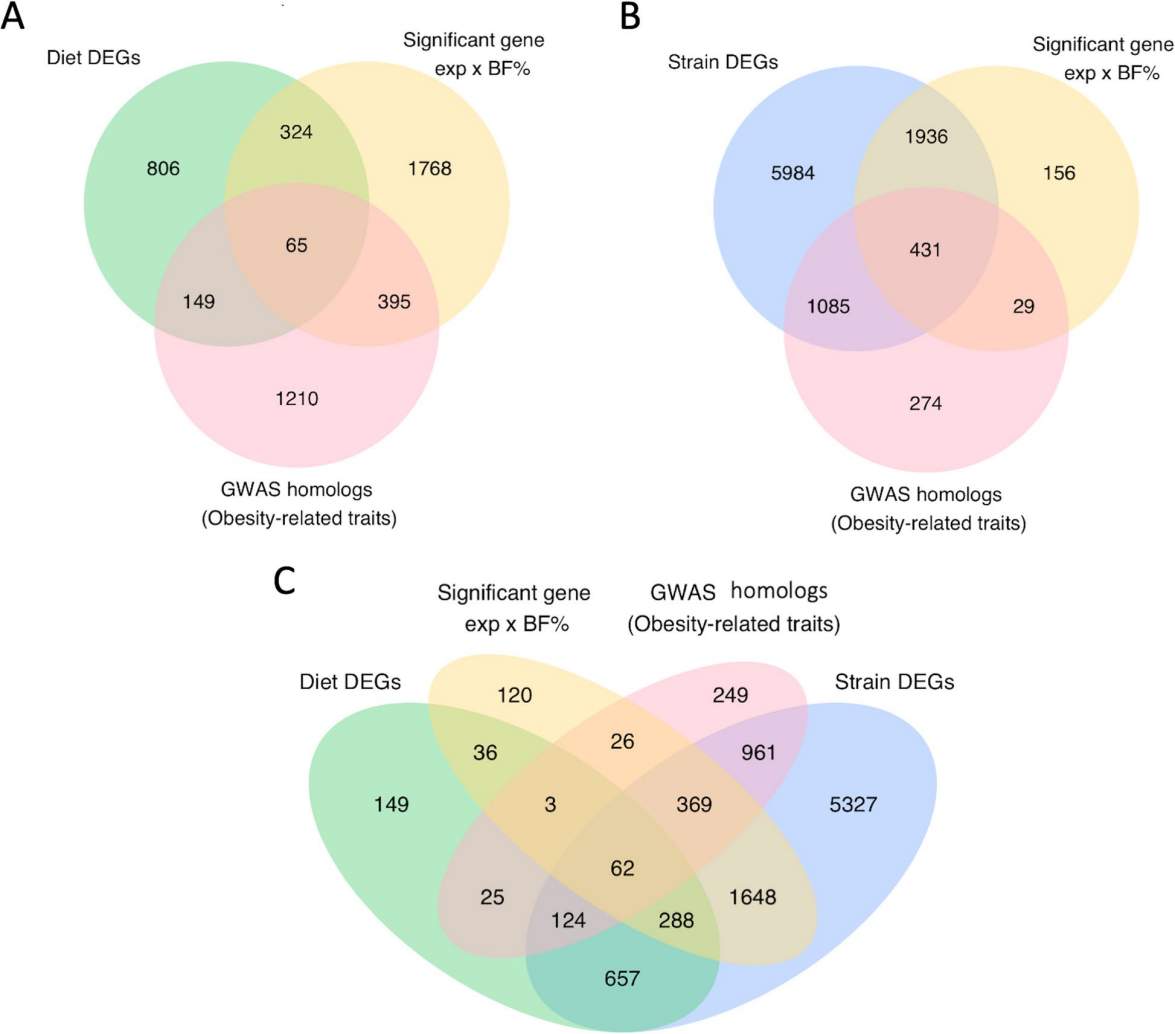
DEGs in the CC were associated with obesity-related traits in humans. Comparisons of differentially expressed genes, genes with expression levels significantly correlated with body fat % (BF%), and genes previously found to be associated with obesity-related traits in the GWAS catalog revealed **A** the number of genes differentially expressed by diet that also had expression levels significantly correlated with body fat % and associated with obesity traits in humans (65), **B** the number of genes differentially expressed by CC strain that also had expression levels significantly correlated with body fat % and associated with obesity traits in humans (431), and **C** the number of genes that fall under all four categories (62). Gene annotation, body fat % correlations, limma statistics, and a subset of related GWAS annotation are shown for the 65 diet DEGs in Supplementary Table 7 and 431 strain DEGs in Supplementary Table 8 (see Additional file 2)

Of the 1344 genes differentially expressed by diet, 214 genes were found to be associated with obesity traits in humans according to the GWAS database; 65 of these 214 genes were also significantly correlated with body fat % in the CC (Fig. 4A; see Supplementary Table 7, Additional file 2). Out of 9436 genes differentially expressed by CC strain, 1516 genes were found to be associated with obesity traits in humans according to the GWAS database, including *Hmga1* and *Irs1*; 431 of these 1516 genes were also significantly correlated with body fat % in the CC (Fig. 4B; see Supplementary Table 8, Additional file 2). By intersecting our lists of genes across multiple analyses, we found 434 differentially expressed genes with expression levels correlated with body fat % in the CC that were associated with obesity traits in humans (Fig. 4C; see Supplementary Tables 7 and 8, Additional file 2), with three genes exclusively differentially expressed by diet, 369 genes exclusively differentially expressed by strain (e.g., *Ide*), and 62 genes differentially expressed by both diet and strain (e.g., *Pik3r1*).

### Differences in diet macronutrient composition had mild effects on broad sense heritability (H^2^) estimates for gene expression levels

To quantify the degree to which genetic variation influences variation in gene expression levels, we calculated broad sense heritability (*H*^2^) for the 11,542 genes used for differential gene expression analysis, which estimates the proportion of phenotypic variation attributed to differences between genetic variation [20]. Using hepatic gene expression as the observed “phenotype” in this study, *H*^2^ was estimated by calculating the intraclass correlation (*r*_I_) and coefficient of genetic determination (*g*^2^) from the between- and within-strain mean square values (MSB and MSW, respectively) derived from linear models. The proportion of variation accounted for by differences between strains can be approximated by *r*_I_ in general, while the calculation of *g*^2^ takes into consideration the additive genetic variance that doubles during inbreeding [18, 20, 47]. Estimates of *H*^2^ based on *g*^2^ calculated using MSB and MSW derived from the “full” additive linear models for the 11,542 genes expressed in the liver used for differential gene expression analysis ranged from −0.056 to 0.983 with a median *g*^2^ of 0.173. To assess whether differences in macronutrient composition (“diet environment”) influenced *H*^2^ by DEG status, *r*_I_ and *g*^2^ summary statistics were calculated for all expressed genes, diet DEGs, and strain DEGs (Table 1); *g*^2^ for diet DEGs ranged from −0.044 to 0.735 with a median of 0.195, while *g*^2^ for strain DEGs ranged from 0.045 to 0.983 with a median of 0.211. For diet-specific *g*^2^, the minimum *g*^2^ values were slightly less than 0, implying that the variation in expression levels for these genes was greater within strains than between strains, but maximum *g*^2^ and median *g*^2^ values were similar both across diets and DEG status. Overall, the distributions of *g*^2^ specifically for the HP and HS diets did not differ significantly neither by the Mann-Whitney test (*W* = 67,447,080, *p* = 0.098) nor the Kolmogorov-Smirnov test (*D* = 0.017, *p* = 0.074), demonstrating that the proportion of variation in gene expression levels attributed to genetic variation stays relatively constant despite differences in macronutrient composition.

**Table 1:** Heritability estimate and diet intraclass correlation summary statistics for all expressed genes and DEGs

Heritability estimate or diet ICC	Mean ± SE	Median (Q1, Q3)	Min	Max
*r*_I_ full — all expressed genes	0.327 ± 0.002	0.295 (0.157, 0.471)	−0.12	0.991
*r*_I_ full — diet DEGs	0.341 ± 0.005	0.327 (0.202, 0.471)	−0.091	0.848
*r*_I_ full — strain DEGs	0.387 ± 0.002	0.348 (0.232, 0.513)	0.087	0.991
*r*_I_ HP— all expressed genes	0.324 ± 0.002	0.305 (0.136, 0.498)	−0.332	0.99
*r*_I_ HP — diet DEGs	0.339 ± 0.006	0.34 (0.179, 0.497)	−0.288	0.899
*r*_I_ HP — strain DEGs	0.388 ± 0.002	0.367 (0.221, 0.545)	−0.194	0.99
*r*_I_ HS — all expressed genes	0.328 ± 0.002	0.313 (0.146, 0.498)	−0.359	0.993
*r*_I_ HS —diet DEGs	0.348 ± 0.006	0.345 (0.203, 0.5)	−0.264	0.887
*r*_I_ HS — strain DEGs	0.389 ± 0.002	0.372 (0.228, 0.539)	−0.234	0.993
*g*^2^ full — all expressed genes	0.218 ± 0.002	0.173 (0.085, 0.308)	−0.056	0.983
*g*^2^ full — diet DEGs	0.221 ± 0.004	0.195 (0.112, 0.308)	−0.044	0.735
*g*^2^ full — strain DEGs	0.26 ± 0.002	0.211 (0.131, 0.345)	0.045	0.983
*g*^2^ HP — all expressed genes	0.223 ± 0.002	0.18 (0.073, 0.331)	−0.142	0.98
*g*^2^ HP — diet DEGs	0.226 ± 0.005	0.205 (0.098, 0.331)	−0.126	0.816
*g*^2^ HP — strain DEGs	0.267 ± 0.002	0.224 (0.124, 0.374)	−0.089	0.98
*g*^2^ HS — all expressed genes	0.224 ± 0.002	0.186 (0.079, 0.331)	−0.152	0.985
*g*^2^ HS — diet DEGs	0.232 ± 0.005	0.208 (0.113, 0.333)	−0.116	0.797
*g*^2^ HS — strain DEGs	0.267 ± 0.002	0.229 (0.129, 0.369)	−0.105	0.985
Diet ICC — all expressed genes	0.055 ± 0.001	0.015 (−0.009, 0.079)	−0.017	0.799
Diet ICC — diet DEGs	0.266 ± 0.003	0.235 (0.172, 0.327)	0.099	0.799
Diet ICC — strain DEGs	0.061 ± 0.001	0.019 (−0.008, 0.089)	−0.017	0.787

Post-diet heritability estimates were calculated from linear models including strain, diet, and week as covariates (*r*_I_ or *g*^2^ “full”) for gene expression of the 11,542 expressed genes used in limma differential gene expression analysis. Diet-specific estimations of broad sense heritability were also calculated accordingly for gene expression levels represented by intraclass correlations (*r*_I_) and coefficients of genetic determination (*g*^2^) for each trait using the MSB and MSW for strain derived from linear models with strain and week as covariates using only data from each experimental diet per model as indicated to assess how different diet “environments” affect heritability. The intraclass correlation for diet (Diet ICC), which is the proportion of the total phenotypic variation that is accounted for by differences between diet, was calculated to compare the proportion of variation in gene expression attributed to diet in general or genetics. Summary statistics were calculated for each group of genes after heritability estimates, and diet ICC were obtained. *g*^2^ accounts for the additive genetic variance that doubles during inbreeding and may be a more appropriate estimate for broad sense heritability in this study. However, both *r*_I_ and *g*^2^ values are presented to facilitate comparisons with other findings in the literature

To quantify the proportion of the total gene expression variation that is accounted for by differences between diet, we next calculated the diet intraclass correlation (ICC) using the diet MSB and MSW values derived from the “full” additive linear models and then calculated summary statistics by DEG status group, i.e., all expressed genes, strain DEGs, and diet DEGs (Table 1). Diet ICC for all expressed genes ranged from −0.017 to 0.799 with a median diet ICC of 0.015. Similarly, diet ICC for strain DEGs ranged from −0.017 to 0.787 with a median of 0.019. Though the maximum diet ICC for diet DEGs (diet *ICC* = 0.799) was similar to the diet ICC maximum values for all expressed genes and strain DEGs (Table 1), the diet DEGs’ minimum (diet *ICC* = 0.099) and median (diet *ICC* = 0.235) estimates were slightly higher, confirming that the proportion of gene expression variation explained by diet differences was modestly increased for diet DEGs.

To investigate the degree to which gene × environmental (diet) effects mediate variation in gene expression relative to genetics and environment, additional linear mixed model analyses with strain, diet, and strain × diet interactions all as random effects were performed for each gene to estimate the relative heritable variation that can be attributed to strain, diet, and strain × diet effects. From the results of these models, we calculated the variance for each of these terms and found that the proportion of heritable variation for gene expression attributed to strain × diet interactions on average was small (2.6%) and remained the same regardless of DEG status (Table 2). For all genes used in differential expression analysis, the largest proportion of heritable variation for gene expression can be attributed to genetic background (strain) on average (30.3%), while the proportions of heritable variation for gene expression attributed to diet (3.9%) and strain × diet interactions (2.6%) were much smaller. As expected, the proportion of heritable variation for gene expression attributed to diet was increased in diet DEGs (18.7%), and the proportion of heritable variation for gene expression attributed to strain was increased in strain DEGs (36.0%).

**Table 2:** Estimating average relative heritable gene expression variation

	CC strain		Diet		CC strain × diet	
	Variance	POV (%)	Variance	POV (%)	Variance	POV (%)
**All expressed genes**	0.069	30.3	0.007	3.9	0.004	2.6
**Diet DEGs**	0.055	26.2	0.033	18.7	0.004	2.6
**Strain DEGs**	0.083	36.0	0.007	3.9	0.004	2.6

To estimate the relative heritable variation that can be attributed to genetics, environment (diet), and gene × environmental effects, linear mixed model analyses with CC strain, diet, and CC strain × diet interactions all as random effects were performed to quantify the proportions of variance (POV) attributed to each term relative to each other for the 11,542 expressed genes used in limma differential gene expression analysis. The mean approximate values for proportion of variance for strain, diet, and interaction were calculated by dividing the variance for each term by the sum of the variance for all terms in the model and multiplied by 100

### Transcriptional co-expression network analysis identified key modules associated with adiposity

Because polygenic obesity is a complex physiological trait, we used a gene co-expression network approach to characterize the effects of strain and diet on expression of groups of related genes in addition to assessment of genes individually, specifically weighted gene co-expression network analysis (WGCNA). WGCNA determines which genes have similar expression profiles using a clustering method based on correlations of gene expression, which identifies the network modules (groups of related genes); measures derived from gene expression correlations influence the strength of connections between genes within the network, where the highly interconnected genes that form modules may be components of biological pathways, helping to bridge the effects of individual genes and resulting phenotypes [45, 106, 107].

**Fig. 5:**
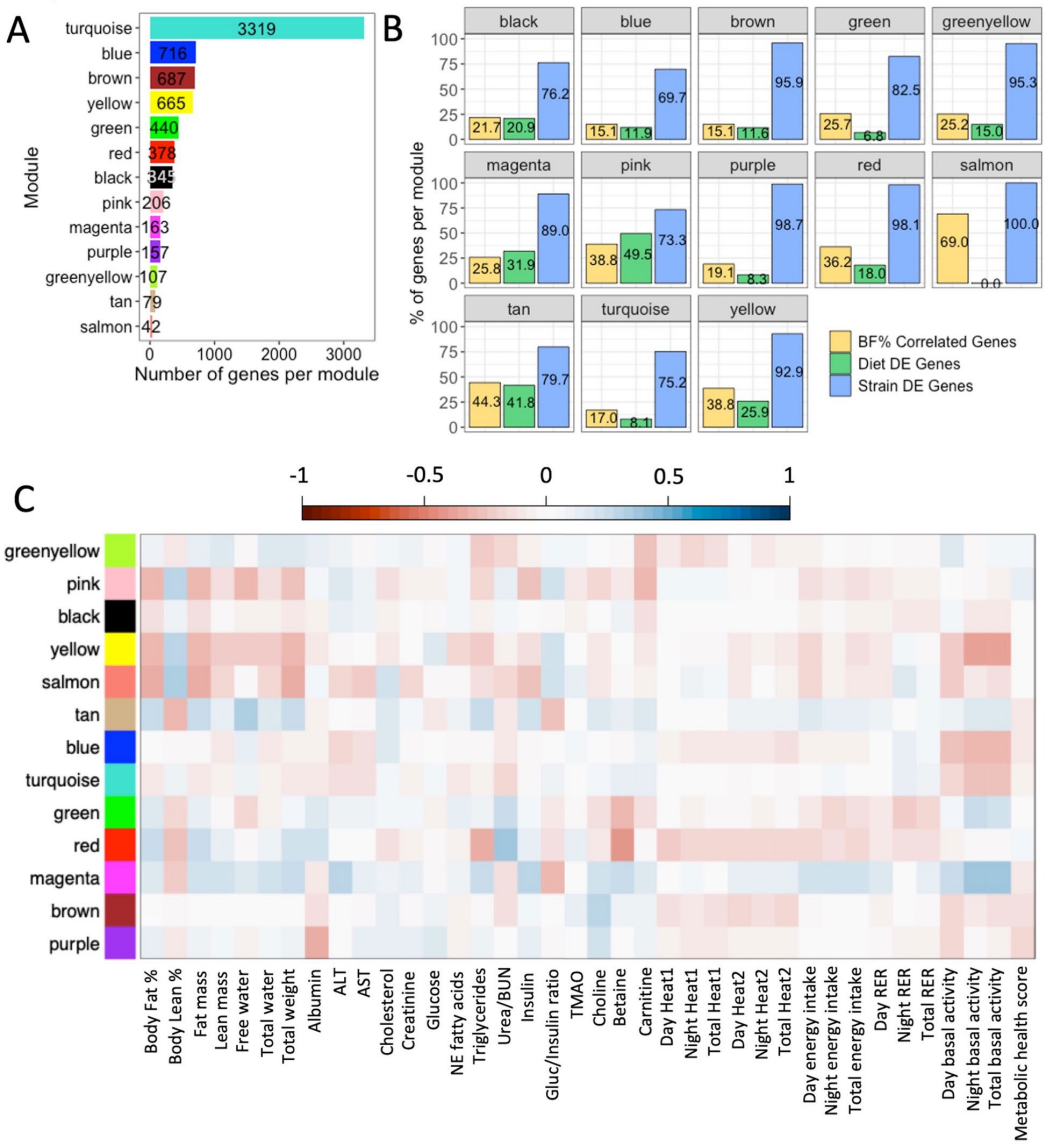
WGCNA identified co-regulated gene modules correlated with phenotypic traits. Using the cleaned and filtered hepatic gene expression data from mice fed the HP diet and mice fed the HS diet, **A** weighted gene co-expression network analysis (WGCNA) identified 13 modules with arbitrarily assigned colors. The 11,542 expressed genes were used to form the modules, which varied widely in terms of the number genes within each module. Gene module assignments are shown in Supplementary Table 9, Additional file 2.** B** Modules demonstrated a wide compositional range in terms of genes with expression levels significantly correlated with body fat % (15.1–69.0%) and differential expression by diet (0–49.5%) but consistently contained a high proportion of genes differentially expressed by CC strain (69.7–100%). **C** The heatmap of Spearman’s correlations between module eigengenes and phenotypic traits measured in the CC mice revealed significant correlations between the pink, yellow, salmon, tan, red, and magenta modules with body fat %. Scale indicates the strength of correlations. Correlation values in **C** are shown in Supplementary Table 10, Additional file 2

Taking a global approach to elucidate the relationship between gene expression and emergent phenotypes, WGCNA was performed using the 11,542 genes expressed in the liver and identified 13 clusters of genes (modules) each assigned an arbitrary color, where the number of genes contained in each module ranged from 42 to 3319 (Fig. 5A, Table 3; see Supplementary Table 9, Additional file 2 and Supplementary Fig. 2, Additional file 1) with varying degrees of connectivity between genes (see Supplementary Fig. 3, Additional file 1 for an example). The percentage of genes significantly correlated with body fat % (15.1–69.0%), and the percentage of DEGs by diet (0–49.5%) showed a wide range of variation in gene numbers across modules, but the percentage of DEGs by CC strain remained consistently high (> 69%) for all modules (Table 3, Fig. 5B); the consistently high presence of strain DEGs in all modules compared to the lower percentage and variation of diet DEGs between modules suggest a stronger effect of CC strain than diet on gene expression. Of the DEGs with expression levels correlated with body fat % and associated with obesity-related traits in humans, the three diet DEGs were each assigned to different modules (black, blue, and pink); the range of strain DEGs per module was 1–106, with the turquoise module containing the highest number of strain DEGs (Table 4). Per module, the range of DEGs differentially expressed by both diet and strain with expression levels correlated with body fat % and also associated with obesity-related traits in humans was 0–19, where most modules contained at least one DEG and yellow contained the most DEGs (Table 4).

**Table 3:** Module gene composition

Module colors	BF% correlated genes	Diet DEGs	Strain DEGs	GWAS obesity traits genes	Total genes
Turquoise	564	268	2497	719	3319
Blue	108	85	499	104	716
Brown	104	80	659	142	687
Yellow	258	172	618	123	665
Green	113	30	363	71	440
Red	137	68	371	74	378
Black	75	72	263	75	345
Pink	80	102	151	43	206
Magenta	42	52	145	27	163
Purple	30	13	155	23	157
Greenyellow	27	16	102	10	107
Tan	35	33	63	20	79
Salmon	29	0	42	11	42

WCGNA identified 13 gene modules each assigned an arbitrary color with the number of genes contained in each module ranging from 42 (salmon) to 3119 (turquoise). Each gene module showed variation in terms of the number of genes with expression significantly correlated with post-diet body fat (BF%), genes differentially expressed by diet, genes differentially expressed by strain, and genes associated with obesity traits in humans according to the GWAS catalog

**Table 4:** DEGs with expression correlated with body fat % associated with obesity in humans

Module colors	Diet DEGs	Gene DE by diet and strain	Strain DEGs
Turquoise	0	9	106
Yellow	0	19	35
Red	0	0	22
Brown	0	1	15
Green	0	1	15
Black	1	2	13
Blue	1	2	8
Pink	1	6	7
Purple	0	0	6
Salmon	0	0	5
Tan	0	3	5
Greenyellow	0	1	1
Magenta	0	0	1

By intersecting lists of genes across multiple analyses, 434 DEGs in the CC were found to have gene expression levels significantly correlated with body fat % and genes associated with obesity traits in humans in the GWAS catalog, with 3 diet DEGs, 369 strain DEGs, and 62 genes differentially expressed by both diet and strain. The number of genes belonging to each category and assigned to the respective module is shown above, with 148 genes not assigned to any module

After establishing the modules, module eigengenes (MEs) were calculated to estimate the average expression profiles of each module, and Spearman’s correlations were performed between MEs and phenotype data from all mice to determine the relationships between the modules and measured phenotypic traits, revealing significant correlations between the pink, yellow, salmon, tan, red, and magenta modules and body fat % (Fig. 5C; see Supplementary Table 10, Additional file 2). Concurrent with ME × phenotype data correlations, modules that were significantly correlated with body fat % had relatively higher percentages of individual genes whose expression levels were significantly correlated with body fat %.

**Fig. 6:**
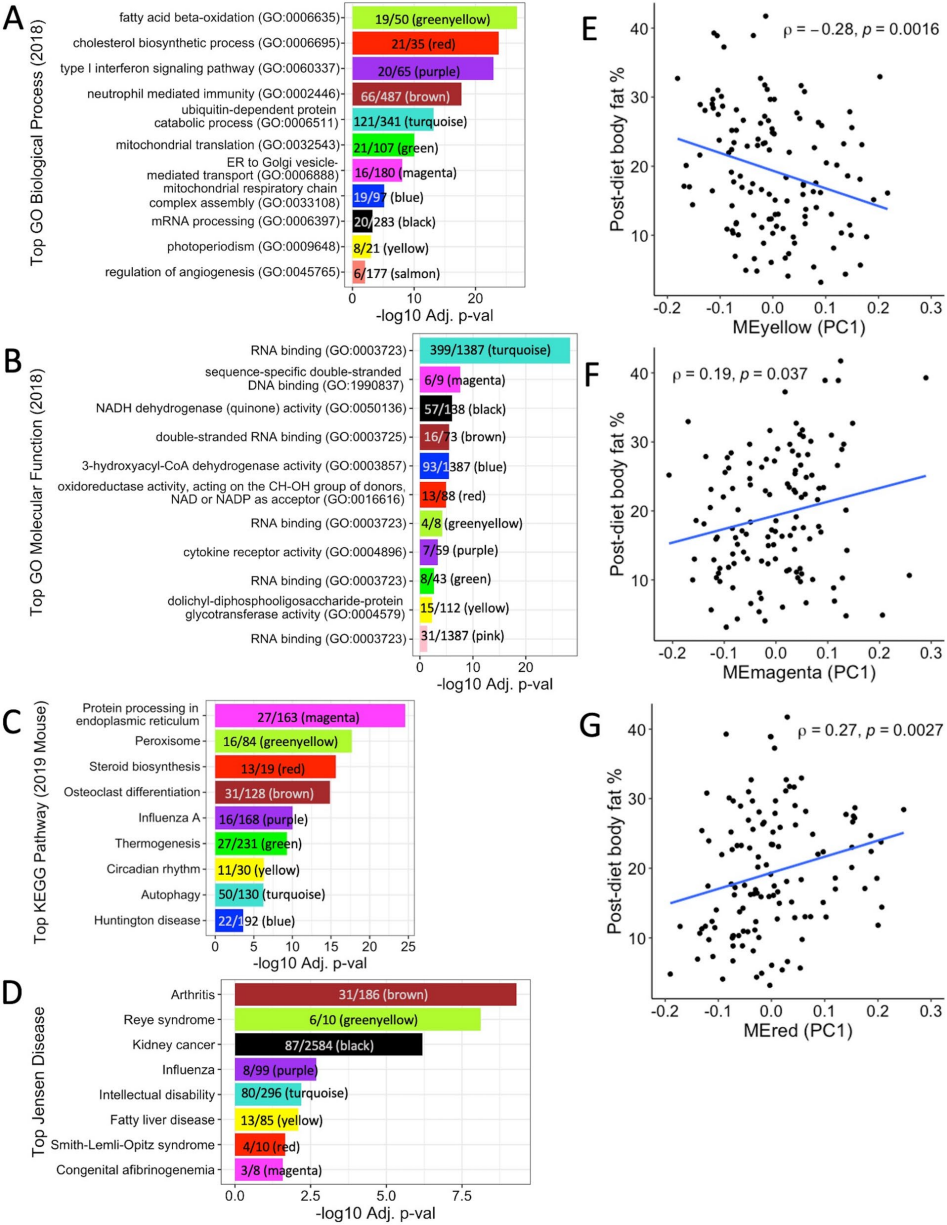
Modules enriched for distinct liver functions were also correlated with body fat %. Enrichr analysis performed using the most recent versions of respective databases identified the top significant enrichment for each module, if available; genes belonging to the tan module did not show any significant enrichment. **A** All modules showed significant enrichment for at least one GO biological process, except for the tan and pink modules. **B** Similarly, all modules showed significant enrichment for at least one GO molecular function, except for the tan and salmon modules. Fewer modules were enriched for **C** KEGG pathways and **D** Jensen diseases. Spearman’s correlations between post-diet body fat % and **E** yellow ME (PC1) (rho = −0.28, *p* = 0.0016), **F** magenta ME (PC1) (rho = 0.19, p = 0.037), and **G** red ME (PC1) (rho = 0.27, *p* = 0.0027) show significant overall associations between average expression profiles of modules identified by WGCNA and body fat %

Because multiple modules were associated with clinical phenotypes (Fig. 5C), we performed enrichment analysis to determine potential mechanisms underlying these associations. Module enrichment varied widely (Table 5), from no enrichments at all (tan) to 419 total enrichments (brown). Figure 6A–D shows the top enrichments for each module if present. Of the modules that were significantly correlated with body fat % in the CC, the tan module showed no enrichments, the pink module showed enrichment for the RNA binding GO molecular function (GO: 0003723) (*p* adj = 0.042), the salmon module showed enrichment for the regulation of angiogenesis (GO: 0045765) (*p* adj = 0.009) and cGMP metabolic process GO biological processes (GO: 0046068) (*p* adj = 0.046), and the magenta, red, and yellow modules showed multiple enrichments for GO biological processes, GO molecular functions, KEGG pathways, and Jensen diseases (Supplementary Figs. 4–6, Additional file 1; Supplementary Table 11, Additional file 2). Genes in the magenta module were significantly enriched for GO terms and KEGG pathways related to endoplasmic reticulum function (Supplementary Fig. 4, Additional file 1); genes assigned to the red module were significantly enriched for GO terms and KEGG pathways involved in steroid, cholesterol, and fatty acid biosynthesis/metabolism (Supplementary Fig. 5, Additional file 1); and genes found in the yellow module were significantly enriched for a variety of functions in terms of GO terms and KEGG pathways, such as photoperiodism, transcription regulation, insulin signaling, and more (Supplementary Fig. 6, Additional file 1).

**Table 5:** Distribution of significant enrichment terms across modules

	GO biological process 2018	GO molecular function 2018	Jensen diseases	KEGG 2019 mouse	Total
Brown	296	25	18	80	419
Turquoise	289	55	1	49	394
Greenyellow	25	11	18	16	70
Red	37	2	3	24	66
Purple	34	2	2	14	52
Yellow	27	11	2	6	46
Magenta	35	4	2	2	43
Black	18	17	3	0	38
Green	24	5	0	8	37
Blue	22	3	0	8	33
Salmon	2	0	0	0	2
Pink	0	1	0	0	1
Tan	0	0	0	0	0

In Enrichr analysis, genes assigned to each module were used to determine whether modules were significantly enriched for functional terms, pathways, or diseases (enrichment terms). Modules varied widely in terms of the number of enrichments for each category, from no enrichments at all (tan) to 419 total enrichments (brown)

### Both diet macronutrient composition and genetic background affected expression of modules containing homologs associated with obesity in humans

The magenta, red, and yellow modules were enriched for biological pathways and correlated with body fat % (Figs. 5C and 6E–G; Supplementary Figs. 4–6, Additional file 1). To determine whether these modules contained DEGs in the CC associated with obesity in humans, the lists of genes assigned to each module were intersected with the list of genes previously found to be associated with obesity traits in humans in the GWAS catalog (Supplementary Table 9, Additional file 2), with examples for these modules shown in Table 6. By intersecting our results across different analyses, DEGs important to obesity in humans were found in biologically relevant modules associated with body fat % in the CC, where the DEG distribution across modules highlighted the larger contribution of differential expression by strain over diet.

**Table 6:** DEGs assigned to enriched modules associated with obesity traits in humans

	Magenta module		Red module		Yellow module	
	Number of DEGs	Genes associated with obesity traits in humans	Number of DEGs	Genes associated with obesity traits in humans	Number of DEGs	Genes associated with obesity traits in humans
**Diet DEGs**	0	NA	0	NA	3	*Fars2*, *Mdfic*, *Abhd15*
**Strain DEGs**	16	*Macrod1*^a^, *Vegfb*, *Serp1*	47	*Fasn*^a^, *Acaca*^a^, *Ppil1*^a^	87	*Nicn1*^a^, *Pnpla7*^a^, *Syne3*^a^, *Clock*^a^
**DEGs by diet and strain**	5	*Uggt1*, *Itih1*, *Serpina6*	12	*Spc24*, *Mipep*, *Cyb5b*, *Dlat*	30	*Fbxo21*^a^, *Brap*^a^, *Mgrn1*^a^

Multiple DEGs in the CC assigned to enriched modules were associated with obesity traits in humans in the GWAS catalog. The number of DEGs for the magenta, red, and yellow modules identified by WGCNA illustrates the larger contribution of differential expression by strain over diet. Examples of genes with human homologs associated with obesity traits are shown for each module, where ^a^ denotes genes that are significantly correlated with body fat % in the CC

**Fig. 7:**
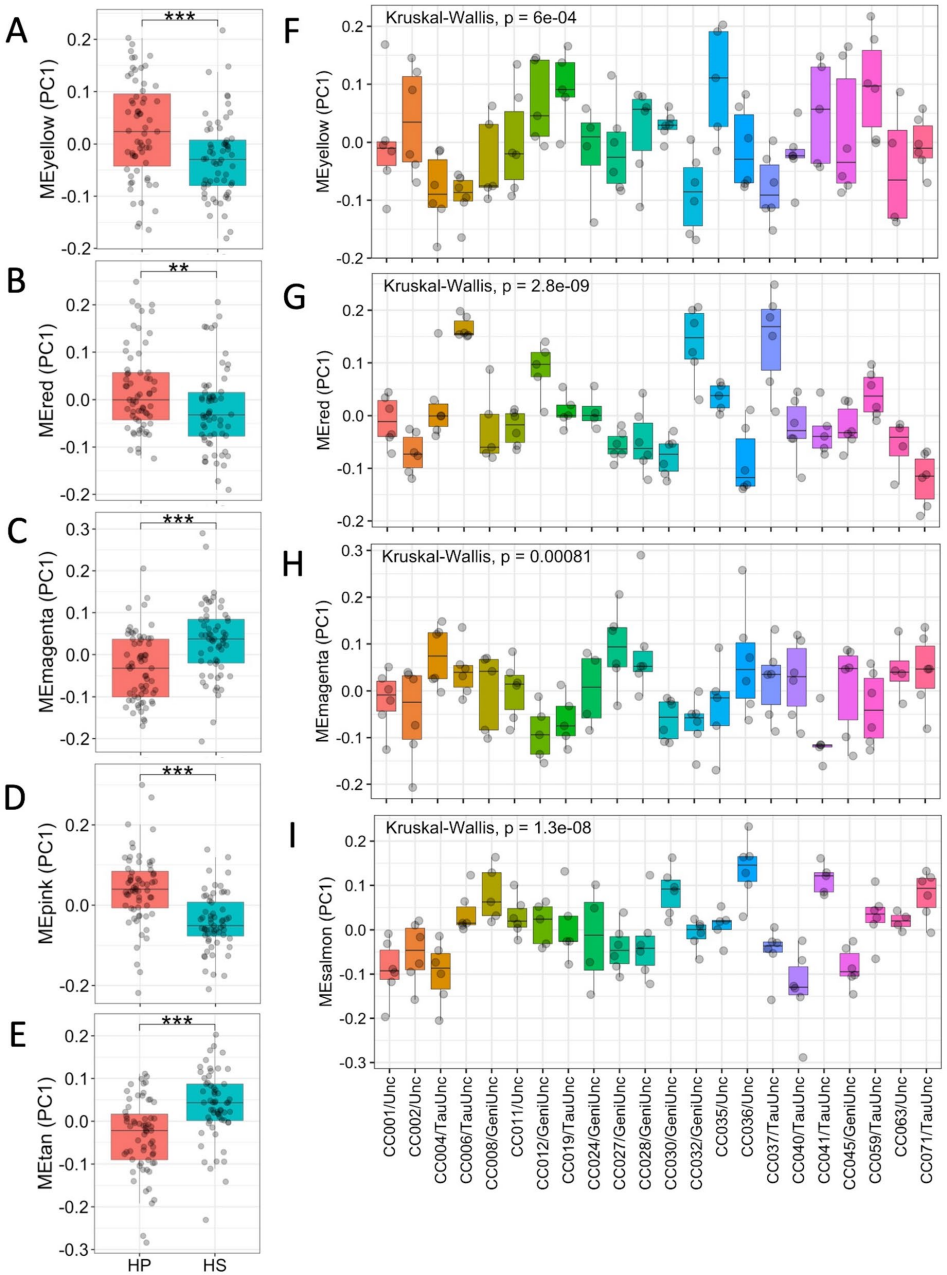
Differences in diet macronutrient composition and genetic background perturb MEs (PC1). Most module eigengene (ME) average gene expression profiles (PC1) significantly correlated with body fat % also significantly differed by diet to different degrees, as ascertained with Wilcoxon ranked-sum tests. The MEs that significantly differed by diet were **A** yellow (*p* < 0.001), **B** red (*p* < 0.01), **C** magenta (*p* < 0.001), **D** pink (*p* < 0.001), and **E** tan (*p* < 0.001), but not salmon (*p* > 0.1). Most module eigengene (ME) average gene expression profiles (PC1) significantly correlated with body fat % also significantly differed by CC strain to different degrees, as ascertained with Kruskal-Wallis tests. The pink and tan MEs did not differ significantly by CC strain (*p* > 0.07), but the MEs for the **F** yellow (*p* = 6.0 × 10^−4^), **G** red (*p* = 2.8 × 10^−9^), **H** magenta (*p* = 8.1 × 10^−4^), and **I **salmon (*p* = 1.3 × 10^−8^) modules differed significantly by CC strain. Points indicate individual calculated ME expression for each mouse, and CC strains are ordered numerically

After finding that gene modules were correlated with body fat % and contained DEGs, we ascertained whether the average gene expression profile of these modules defined by their ME first principal components (PC1) differed by diet and/or strain. Wilcoxon ranked-sum test of the PC1 between mice fed the HP and HS diets for each module (Fig. 7A–E) revealed significant differences by diet for the yellow, red, magenta, pink, and tan modules (*p* < 0.01), but not the salmon module (*p* > 0.1). Interestingly, when the Kruskal-Wallis test was performed to determine whether PC1 differed by strain for each module (Fig. 7F–H), PC1 significantly differed by strain for the yellow, red, magenta, and salmon modules (all *p* ≤ 8.1 × 10^−4^), but not the pink nor tan modules. Of the modules with MEs significantly correlated with body fat %, the yellow, red, and magenta modules exhibited differences by both macronutrient composition and CC strain.

Relating module MEs and body fat %, Spearman’s correlations performed between MEs and body fat % for the yellow, red, and magenta modules using data from all samples revealed a significant negative correlation between body fat % and the yellow module (rho = −0.28, *p* = 0.0016) and significant positive correlations between body fat % and the magenta (rho = 0.19, *p* = 0.037) and red (rho = 0.27, *p* = 0.0027) modules (Fig. 6E–G). Given the many enrichments in biological pathways found and significant differences in MEs by diet and CC strain for these three modules, Spearman’s correlations were performed between MEs and body fat % by diet for each module to determine whether the relationship between MEs and body fat % remained consistent across different diets for enriched modules. The correlation between expression of the yellow module and body fat % was significant and negative for the HS diet only, while the correlation between expression of the magenta module and body fat % was significant and positive for the HS diet only (Supplementary Fig. 7, Additional file 1). Unlike the yellow and magenta modules where the correlations between MEs and body fat % were only significant for the HS diet, the correlation between the red ME and body fat % remained significant and consistently positive for both diets (Supplementary Fig. 7, Additional file 1). In summary, Spearman’s correlations performed between MEs and body fat % by diet for biologically relevant modules illustrated alterations in the direction and magnitude of associations between module MEs and body fat % depending on diet for the yellow and magenta modules, in contrast to the red module where the direction and magnitude of associations between module MEs and body fat % for the red module remained consistent regardless of diet, demonstrating the modules’ different responses to diet.

## Discussion

Obesity is a complex and heterogeneous disease whose development is caused by numerous biological factors, particularly genetics, diet, and gene expression. Though long established that obesity results from a chronic imbalance between energy intake and expenditure at a fundamental level, our understanding of exactly how diet and genetics interact to influence gene expression and how gene expression regulates the development of obesity remains to be fully elucidated. Because the liver regulates metabolism of macronutrients, cholesterol, and triglycerides, we measured hepatic gene expression in the CC to gain insight of how diet and genetic background impact obesity and related obesity-related traits. Correlations performed between hepatic gene expression levels and post-diet phenotype data revealed 2552 genes whose expression levels were significantly correlated with body fat % in the CC, some which were negatively correlated such as *ApoM* and *Fmo3*, while others were positively correlated such as *Aldh1a1* and *Adipor2*. *ApoM* encodes a membrane-bound apolipoprotein associated with high-density lipoproteins, low-density lipoproteins, and triglyceride-rich lipoproteins; secreted through the plasma membrane, alipoprotein M is involved in lipid transport [99]. In the mouse, leptin the “satiety” hormone and leptin receptor are essential for expression of *ApoM*, but excess concentrations of leptin inhibited *ApoM* mRNA expression in a dose-dependent manner in the human hepatoma cell line HepG2, suggesting that leptin may mediate *ApoM* expression [55]. Although FMO3 is more well-known for its role in preventing trimethylaminuria (fishy odor syndrome) in humans [92], FMO3 also functions as a drug-metabolizing enzyme to catalyze the NADPH-dependent oxygenation of various molecules including therapeutic drugs and dietary compounds [65]. Intriguingly, studies in the mouse have suggested additional roles for FMO3 in health and disease, such as modulating cholesterol metabolism [96], glucose, and lipid homeostasis [78], and as a target for downregulation by insulin [58]. Since adipocyte secretion of leptin and insulin occurs in proportion with the volume of adipose tissue under “normal” circumstances, this may partially explain the negative correlations between body fat % and expression of *ApoM* and *Fmo3*.

In the current study, the hepatic gene expression levels of *Aldh1a1* and *Adipor2* were positively correlated with body fat %. *Aldh1a1* encodes the protein aldehyde dehydrogenase 1 family, member A1 (ALDH1A1), also known as retinaldehyde dehydrogenase 1 (RALDH1), which is a prominent enzyme in the oxidative pathway of alcohol metabolism. However, various studies in mice have shown that ALDH1A1 also modulates hepatic gluconeogenesis and lipid metabolism through its role in retinoid metabolism [39], and upregulation of ALDH1A1 is associated with reduced adiponectin expression in adipose tissue after high-fat diet feeding [44]. Furthermore, mice without ALDH1A1 are resistant to diet-induced obesity, and inhibition of ALDH1A1 in mice suppresses weight gain [27, 28], which is consistent with our finding and illustrates the potential for ALDH1A1 as a drug target for obesity prevention or treatment. *Adipor2* encodes adiponectin receptor 2 which interacts with adiponectin to mediate fatty acid oxidation and glucose uptake [103]. An agonist of adiponectin receptor 2, the adipokine adiponectin, is inversely correlated with body fat mass and visceral adiposity in humans, though the mechanisms of how adiponectin’s interactions with its receptors to elicit antidiabetic, anti-atherogenic, and anti-inflammatory effects are not fully understood [63].

After confirming the relationship between expression of genes related to obesity and body fat % in the CC, we investigated the effects of genetic background (strain) and diet on hepatic gene expression levels. Similar to adiposity and the obesity-related traits examined in our previous study [101], genetic background had a far stronger effect on hepatic gene expression than diet, as shown by the overwhelmingly larger number of significant DEGs by strain (9436) compared to the number of DEGs by diet (1344). Interestingly, gene expression of 28.9% of diet DEGs was significantly correlated with adiposity (389/1344) compared to 25% of strain DEGs (2367/9436). Of the top 20 most significant diet DEGs identified in the CC, carbamoyl-phosphate synthase 1 (*Cps1*), isovaleryl-CoA dehydrogenase (*Ivd*), neuropilin 1 (*Nrp1*), and pyruvate kinase L/R (*Pklr*) were previously found to be associated with obesity traits in humans [38, 51, 69, 72, 108], but only one of the top 20 most significant strain DEGs was associated with at least one obesity trait in humans, namely glycerophosphodiester phosphodiesterase domain containing 3 (*Gdpd3*) [108].

Gene enrichment analysis of DEGs revealed different trends between DEGs by diet compared to strain. DEGs by diet showed enrichment for KEGG pathways and Gene Ontology (GO) biological processes related to numerous types of metabolism, amino acid synthesis, and nonalcoholic fatty liver disease, whereas DEGs by strain showed enrichment for cell function pathways, type 1 diabetes, and fatty acid metabolism. Like KEGG pathway enrichment, GO term enrichment for cellular components and molecular functions also showed distinct differences between DEGs by diet compared to strain; DEGs by diet showed enrichment for multiple cellular components related to the mitochondrion, endoplasmic reticulum, and cell membrane, while DEGs by strain showed enrichment for cellular components related to the cell membrane, extracellular components, and cell surface. In terms of molecular functions, DEGs by diet showed enrichment for metabolism and binding for nutrients and small molecules such as cofactor binding, vitamin B6 binding, catalytic activity, and electron transfer activity, while DEGs by strain showed enrichment for binding related to general cell and tissue functions, such as extracellular matrix, collagen, signaling receptor, and fibronectin binding. The culmination of our results suggests that generally, diet alters gene expression for “acute” metabolic processes sensitive to environmental changes, but genetic background more heavily influences overall “essential” cellular function.

Having identified genes with expression strongly influenced by diet or strain, we used the GWAS catalog as a guide to highlight clinically important genes found in our study by determining which DEGs may be most relevant to obesity-related traits in humans. The comparison between DEGs in the CC and genes in the GWAS catalog revealed that 65 diet DEGs and 431 strain DEGs correlated with body fat % in the CC have previously been identified as associated with obesity-related traits such as body fat distribution, BMI, waist-hip ratio, weight, and fat body mass in humans. One caveat regarding the number of DEGs in the CC found to be associated with obesity-related traits in humans is that our study focused only on gene expression in the liver of CC mice, while the genes listed in the GWAS catalog associated with obesity traits include candidates found in multiple tissue types; thus, including gene expression from additional tissue type such as brain or adipose tissue could yield additional candidate genes. Nonetheless, we identified genes expressed in the liver whose expression levels were either under genetic regulation, influenced by diet, or correlated with body fat %, which were also clinically important in humans using the CC panel as a model for obesity, which enabled the use of genetic “replicates” with high genetic diversity so that the results from this study are additive in scope.

In our list of genes whose gene expression levels were significantly correlated with body fat % that have previously been associated with obesity-related traits in humans, some genes exclusively differentially expressed by diet found in our current study include increased sodium tolerance 1 homolog (*Ist1*) [32], chromodomain protein, Y chromosome-like (*Cdyl*) [87], and NIPBL cohesin loading factor (*Nipbl*) [87], while genes exclusively differentially expressed by strain were lysophospholipase-like 1 (*Lyplal1*) [22, 38, 49, 69, 87, 94], leucine-rich repeat (in FLII) interacting protein 1 (*Lrrfip1*) [67], and neurotrophic tyrosine kinase, receptor, type 2 (*Ntrk2*) [1, 38, 69, 108]. Lastly, genes differentially expressed by both strain and diet include F-box protein 21(*Fbxo21*) [38, 69, 108], alanyl-tRNA synthetase (*Aars*) [38, 108], and BRCA1-associated protein (*Brap*) [32, 88, 98]. Our findings highlight which candidate genes previously described in the literature have the highest potential for successful future validation studies.

Using the between- and within-strain mean square values derived from linear models, we calculated *H*^2^ estimates to quantify the degree to which genetic variation affects hepatic gene expression level variation. For the 11,542 genes included in our analysis, the range of coefficient of genetic determination (*g*^2^) was broad as expected (*g*^2^ = −0.056–0.983), but the median was lower than anticipated (*g*^2^ = 0.173) given the strong effect of strain on the expression of most genes. Median *H*^2^ estimates by DEG status increased slightly but not drastically (diet DEG *g*^2^ = 0.195, strain DEG *g*^2^ = 0.211), while *H*^2^ estimates remained similar, suggesting that differences in macronutrient composition did not have a large impact on hepatic gene expression in this study. Upon examination of the relative heritable variation that can be attributed to strain, diet, and strain × diet effects for all genes, the largest proportion of heritable variation for gene expression can be attributed to genetic background (strain) on average (30.3%), while the proportions of heritable variation for gene expression attributed to diet (3.9%) and strain × diet interactions (2.6%) were much smaller, which reaffirms the strong effect of strain on gene expression relative to diet and strain × diet effects. However, one caveat of these approximations is that increasing the sample size would provide a better estimation of the relative heritable variation since the number of mice per strain per diet is relatively low, so the estimation of strain × diet effect may not be precise.

Since obesity is a complex trait regulated by multiple genes, we used a gene co-expression network approach including the 11,542 expressed genes to find groups of genes that are similarly regulated by diet or strain and identified 13 gene modules comprised of a wide number of genes from 42 to 3319. Consistent with our DEG analyses, all modules were comprised largely of genes that were strain DEGs (> 69%), while the proportion of diet DEGs (0–49.5%) and genes with expression significantly correlated with body fat % (15.1–69.0%) varied much more widely, illustrating the variable effect of diet on gene expression compared to genetic background. Spearman’s correlation of the MEs for identified modules with phenotypic data revealed six modules related to body fat %: tan, pink, salmon, magenta, red, and yellow. The MEs for all of these modules differed significantly by diet, except for the salmon module, suggesting that differences in diet macronutrient composition induce changes in gene expression for entire groups of genes. Similar to diet, the MEs for most of the modules also differed significantly by strain, except for the pink and tan modules. However, it is important to note that the ME variation within each strain appeared much higher for these two modules than the magenta, red, and salmon modules, an observation shown through the ability of utilizing genetic “replicates” with high genotypic and phenotypic diversity that is inherent to the CC; in fact, increasing the number of “replicates” would enhance the ability to find significant strain-by-diet differences. Thus, we show that both diet and strain may strongly affect hepatic gene expression, and that the CC can be used to interrogate the sources of inter-individual variation that underlies the variable response to diet observed in humans and mice.

Enrichment analysis performed using the lists of genes assigned to each module allowed us to assess which modules identified in the CC may be most biologically relevant to obesity and human health. Of the six modules whose MEs were significantly correlated with body fat %, the number of enrichment terms were few to none for the salmon, pink, and tan modules, but the magenta, red, and yellow modules were significantly enriched for numerous functional pathways, biological processes, and/or diseases. For example, the magenta module was enriched for pathways related to endoplasmic reticulum (ER) function and contained 163 genes total, with 16 strain DEGs and five DEGs by both diet and strain associated with at least one obesity trait in humans. Two DEGs associated with obesity in humans from the magenta module that merit further study are stress-associated endoplasmic reticulum protein 1 (*Serp1*) and UDP-glucose glycoprotein glucosyltransferase 1 (*Uggt1*). *Serp1* participates in the metabolism of proteins in the ER by protecting target proteins against degradation [102] and was differentially expressed by strain in the CC. Similarly, *Uggt1* encodes the enzyme UDP-glucose:glycoprotein glucosyltransferase (UGT), which is also located in the lumen of the ER and provides quality control for protein transport by selectively enabling misfolded glycoproteins to rebind calnexin, resulting in either the proper folding of the glycoprotein or exposure to degradation enzymes if proper folding fails to occur [15]; *Uggt1* was differentially expressed by both diet and strain in the CC. Studies have demonstrated that hepatic ER stress induced by obesity can lead to the development of hepatic insulin resistance and gluconeogenesis, likely through the activation of the JNK pathway [40, 62, 105]. Our findings reaffirm the association between obesity and alterations in hepatic gene expression related to ER function, suggest potential candidate genes for future study in relation to patient screening for diabetes risk, and provide a link between diet, five hepatic ER genes, obesity, and insulin resistance.

Focusing on nine major genes pivotal to insulin signaling expressed in the liver of CC mice, the expression levels of six genes were significantly correlated with body fat % (*Ide*, *Insig1*, *Insr*, *Irs2*, *Jak1*, and *Pik3r1*), while six genes were only differentially expressed by strain (*Hmga1*, *Ide*, *Insig1*, *Insig2*, *Irs1*, and *Jak1*) and two genes were differentially expressed by both strain and diet (*Irs2* and *Pik3r1*). Although all nine genes except *Jak1* were assigned to a module in our network analysis, only *Insig1*, *Insig2*, and *Irs2* were found in the enriched modules correlated with body fat % (magenta, red, or yellow). Assigned to the red module, *Insig1* (insulin-induced gene 1) illustrates one pathway that insulin signaling regulates to alter lipid metabolism in both mice and humans [61]. In the livers of transgenic mice, overexpression of the INSIG1 protein reduces insulin-stimulated lipogenesis by inhibiting processing of sterol regulatory element-binding proteins (SREBPs) in the ER, membrane-bound transcription factors that activate lipid synthesis [17]. In humans, *INSIG1* variants have been shown to influence obesity-related hypertriglyceridemia [83]. Two genes crucial to insulin signaling that were assigned to the yellow module were *Insig2* (insulin-induced gene 2) and *Irs2* (insulin receptor substrate 2). Similar to *Insig1*, *Insig2* obstructs processing of SREBPs by binding to SREBP cleavage-activating protein in the ER, which results in blockage of cholesterol synthesis [100]. Genetic variants in *INSIG2* (rs75666605) have been associated with severe obesity in a North Indian human population [68] and increased blood pressure and triglyceride levels in Brazilian obese patients [60]. Differentially expressed by both strain and diet, IRS2 is a vital mediator of insulin signaling since it acts as an immediate downstream substrate of insulin receptors and activates a cascade of serine-protein kinases to modulate numerous metabolic processes [2, 14]. In mice, conditional knockout of *Irs2* led to increased appetite and insulin resistance that progressed to diabetes [48] and lower levels of thyroid hormones [34]. In summary, our findings help explain the influences of genetic background and dietary macronutrient composition on clinically significant genes involved in insulin response relative to obesity development.

For future studies, investigating the transcriptome and epigenome of both adipose tissue and hepatic tissue together would further clarify the genetic and dietary mechanisms that drive the cross talk between tissue types to modulate energy balance and insulin response in the context of obesity development. If possible, integrating microbiome data would provide yet another “piece of the puzzle” for the elucidation of how genetic and environmental factors interact in the development of obesity. Nonetheless, our findings show that both variation in genetic background and diet can strongly influence hepatic gene expression of both individual genes and groups of related genes relevant to obesity.

## Conclusions

This study determined the relationship between genetics and macronutrient composition on hepatic gene expression relative to obesity. To relate adiposity and obesity-related traits to hepatic gene expression, correlations were performed using phenotype data and microarray data, revealing 2552 genes whose expression levels were significantly correlated with adiposity. In general, the effect of strain was much stronger than diet on hepatic gene expression as demonstrated by differential gene expression analysis which found over 9000 genes differentially expressed by strain compared to 1344 genes differentially expressed by diet. Interestingly, diet differentially expressed genes (DEGs) were enriched for many biological pathways associated with substrate metabolism, whereas strain DEGs were enriched for pathways less sensitive to environmental perturbations. Because common obesity is caused by multiple genes, weighted gene co-expression network analysis (WGCNA) was performed to identify clusters of related genes grouped into “modules.” Multiple gene modules were found that differed in average expression by both diet and strain, where three of the gene modules were correlated with adiposity and enriched for biological pathways related to obesity development. By combining all the analyses above and searching in the genome-wide association studies (GWAS) catalog, the list of obesity candidate genes found via GWAS in humans can be narrowed down to increase the success of future functional validations studies. Furthermore, we demonstrated that both strain and diet influence expression of individual genes as well as the expression for groups of related genes. By integrating phenotype data into our analysis, we found both individual genes and gene modules expressed in the liver that were related to adiposity and other clinical traits. This work sheds light on one way that genetic background and diet influence adiposity, where the identification of genes expressed in the liver related to adiposity provides concrete preliminary suggestions of specific “intermediary” mechanisms that bridge genetics and diet with obesity such as insulin signaling, which may be validated in future studies and contribute to the field of precision nutrition.

## Methods

### Animals, husbandry, diets, and phenotyping

Details on the origin, housing, husbandry, treatment of the CC mice, diet compositions, and phenotyping have been described previously [101]. Briefly, female mice from 22 CC strains (total *n* = 204) were obtained from the UNC Systems Genetics Core Facility in 2016 and put on either a high-protein (*n* = 102) or high-fat high-sucrose (*n* = 102) diet for 8 weeks followed by analysis of body composition, metabolic rate, and physical activity. After 8 weeks on experimental diets, mice were euthanized following a 4-h fast for the collection of blood and liver tissue. Subsequently, circulating cholesterol, triglyceride (TG), glucose, albumin, creatinine, urea/BUN, aspartate transaminase (AST), and alanine transaminase (ALT) levels were quantified using the Cobas Integra 400 Plus (Roche Diagnostics, Indianapolis, IN), according to manufacturer’s instructions. Circulating insulin was measured using an ultrasensitive mouse insulin ELISA (ALPCO Diagnostics, Salem, NH) per manufacturer’s instructions. Trimethylamine N-oxide (TMAO), choline, betaine, and carnitine were quantified using liquid chromatography-mass spectrometry (LC-MS) methods as described with modifications [95]. Metabolic health scores were calculated using measurements of several metabolic risk factors (circulating glucose, insulin, glucose/insulin ratio, cholesterol, triglycerides, and body fat %) to approximate overall metabolic health [101].

### Microarray analysis for identification of gene expression levels associated with post-diet traits and differentially expressed genes in liver tissue

Methods of RNA extraction from livers and evaluation of RNA integrity were performed as previously described [8]. Randomly selecting 3 mice per stain per diet for microarray analysis, high-quality RNA was available from livers of 127 of the 204 CC mice and hybridized to Affymetrix Mouse Gene 2.1 ST 96-Array Plate using the GeneTitan Affymetrix instrument (Affymetrix, Inc., Santa Clara, CA) according to standard manufacturer’s protocol. The robust multiarray average (RMA) method was used to estimate normalized expression levels of transcripts (median polish and sketch-quantile normalization) using the *affy* R package [23]. The quality of sample arrays was then assessed using the R package arrayQualityMetrics [37] for outlier detection using 3 methods: distance between arrays/principal component analysis, computation of the Kolmogorov-Smirnov statistic *K*_a_ between each array’s intensity distribution and the intensity distribution of the pooled data to compare individual array intensity to the intensity of all arrays, and computing Hoeffding’s statistic *D*_a_ to check individual array quality. Sample arrays identified as outliers by all three methods were removed, i.e., a sample array was removed if all three methods indicated that it was an outlier, leaving 123 out of 127 arrays for analysis (Supplementary Table 12, Additional file 2).

Probes and transcript cluster IDs (TC IDs) were first filtered as described [70], resulting in the total number of 24,004 unique probes post-filter corresponding to 23,626 genes. Next, TC IDs were kept for analysis if their median expression was above the mean of all TC ID medians or if their median expression was above the mean of all TC ID medians in over 12.5% of samples, based on the assumption that by chance, one of the 8 founders may be contributing low/no expression alleles. For TC IDs associated with the same gene, the TC ID with the highest expression was selected to represent that gene so that each gene was represented by a unique TC ID for analysis, resulting in 11,542 TC IDs (genes) used for differential gene expression analysis and correlations between gene expression levels and phenotype data.

After filtering TC IDs and arrays for quality, calculations of multiple biweight midcorrelations (bicor) and their corresponding Student correlation *p*-values were performed for the unique TC IDs corresponding to 11,542 genes using the bicorAndPvalue function from the weighted gene co-expression network analysis (WGCNA) R package [45] to ascertain which genes’ expression in the liver was correlated with post-diet traits. Next, differential gene expression analysis was performed using the linear models for microarray analysis (limma) R package version 3.6.1 [73] and methods described [66] to find genes that were significantly differentially expressed by diet or CC strain. Genes with a Benjamini-Hochberg (BH)-adjusted *p*-value < 0.05 were designated as differentially expressed (DE). The Kyoto Encyclopedia of Genes and Genomes (KEGG) pathway and gene ontology (GO) enrichment analyses were performed using the *kegga* and *goana* functions in limma for differentially expressed genes with the false discovery rate (FDR) cutoff set to 0.05.

### Broad-sense heritability estimates and diet intraclass correlations of hepatic gene expression levels

Broad-sense heritability (*H*^2^) estimates and the intraclass correlations (ICC) for diet were calculated as described previously [101] for the 11,542 genes used in limma analysis to assess the degree of influence on gene expression variation from genetics (strain) and diet, respectively. *H*^2^ was estimated by calculating the intraclass correlation (*r*_I_) and the coefficient of genetic determination (*g*^2^) using mean square between (MSB) strains and mean square within (MSW) strains values derived from linear regression analysis [20]. The following linear models were fit using the *lm* function and implementing Satterthwaite approximations on the output of *lm* as described [54] to obtain MSB and MSW values for *r*_I_ and *g*^2^ calculations: (1) a “full” additive model with strain, diet, and week (mouse “batch”) as variables fitted with gene expression data from both experimental diets, (2) a “HP” additive model including strain and week as variables fitted with gene expression data from only mice fed the HP diet, and (3) a “HS” additive model including strain and week as variables fitted with gene expression data from only mice fed the HS diet. *H*^2^ estimates derived from models fitted with data from all mice post-diet compare the contribution of genetics (strain) and diet overall to heritable gene expression level variance, while diet-specific *H*^2^ estimates were calculated to discern differences in heritability affected by differences in macronutrient composition. The diet ICCs were calculated using the mean square between (MSB) diets and mean square within (MSW) diets derived from the “full” additive linear model described above. Additional linear mixed model analyses with strain, diet, and strain × diet interactions as all random effects were performed for each gene to estimate the relative heritable variation in gene expression that can be attributed to strain, diet, and strain × diet effects.

### Weighted gene co-expression network analysis (WGCNA)

The WGCNA R package was used to identify modules for the 11,542 expressed genes used in microarray analysis of differentially expressed genes since complex traits often result from changes in expression of multiple genes. Expression data from the 123 non-outlier sample arrays were used to detect modules, which are groups of highly correlated genes with similar connection strengths [24, 106]. The soft threshold was chosen by running the *pickSoftThreshold* function to determine the best fit to a scale-free topology, and beta was set to 5 because it was the lowest power value where the *R*^2^ value crossed the 0.9 threshold for approximate scale-free topology and connectivity measures implicated the possibility of finding highly connected genes. The *blockwiseModules* function was run to construct the unsigned network in one block, calculate an adjacency matrix with Pearson correlations, calculate the topological overlap matrix (TOM) using the signed method, cluster genes using the default average linkage hierarchical clustering, and establish modules by the dynamic hybrid tree cut method [45]. Next, the *mergeCloseModules* function was used to merge closely related and highly correlated modules. Module eigengenes were calculated, and Spearman’s correlations were performed between module eigengenes and measured phenotypes. KEGG pathway enrichment and gene ontology analyses were performed on genes within each module using Enrichr as described [70] to see which modules contained genes associated with biological function or diseases. Cytoscape [77] was used to generate a visualization of the relationship between genes within a module, using the magenta module as an example.

### Human GWAS catalog analysis

Entries in the EMBL-EBI Human GWAS catalog v1.0.2 accessed in 2021 were indexed to matching mouse genes [4] to compare the DEGs found in the CC with homologous genes in humans. Human gene symbols from the “MAPPED_GENE” catalog column (described here: https://www.ebi.ac.uk/gwas/docs/methods/curation) were matched against mouse gene symbols after case normalization, white space removal, and, in the case of multiple mapped genes, delimiter separation.

### Additional statistical analyses

All statistical analyses were performed in R (v.3.6.1) [71]. Diet or strain effects on module eigengenes were assessed using the two-group Mann-Whitney *U* (Wilcoxon rank) test or Kruskal-Wallis statistical test, respectively. The Mann-Whitney *U* (Wilcoxon rank) test and Kolmogorov-Smirnov test were performed to test whether the distributions of diet-specific *H*^2^ estimates (*g*^2^) differed significantly. In general, *p*-values were adjusted using the Benjamini-Hochberg (BH) method where indicated.

## Supplementary Information

**Additional file 1: Supplementary Figure 1.** Analyses framework and main results. **Supplementary Figure 2.** Dendrogram WGCNA identifies gene co-regulated modules. **Supplementary Figure 3.** Connectivity for genes assigned to the magenta module. **Supplementary Figure 4.** Significantly enriched pathways and ontologies for the magenta module. **Supplementary Figure 5.** Significantly enriched pathways and ontologies for the red module. **Supplementary Figure 6.** Significantly enriched pathways and ontologies for the yellow module. **Supplementary Figure 7.** Correlations between the magenta, red, and yellow MEs with body fat % by diet.

**Additional file 2: Supplementary Table 1.** All genes significantly correlated with post-diet body fat % (2,552). **Supplementary Table 2.** Significant correlations between phenotypes and the top 30 genes with expression levels most strongly correlated with post-diet BF%. **Supplementary Table 3.** All genes with significant differential expression by diet (1,344). **Supplementary Table 4.** Genes differentially expressed by diet in the CC were significantly enriched for 20 KEGG pathways and 187 GO terms. **Supplementary Table 5.** All genes with significant differential expression by strain (9,436). **Supplementary Table 6.** Genes differentially expressed by strain in the CC were significantly enriched for 13 KEGG pathways and 163 GO terms. **Supplementary Table 7.** 65 DE genes by diet associated with obesity traits in humans are also significantly correlated with body fat %. **Supplementary Table 8.** 431 DE genes by strain associated with obesity traits in humans are also significantly correlated with body fat %. **Supplementary Table 9.** Module gene assignments. **Supplementary Table 10.** Spearman’s correlations between module eigengenes (MEs) and phenotypic traits. **Supplementary Table 11.** All significant enrichments for magenta, red, and yellow modules. **Supplementary Table 12.** Population of mice used in microarray analysis.

## Data Availability

The phenotype dataset analyzed during the current study is available in the Mouse Phenome Database (https://phenome.jax.org, RRID:SCR_003212, Bennett4). The microarray dataset generated and analyzed during the current study has been deposited in NCBI’s Gene Expression Omnibus and is accessible through GEO series accession number GSE185547 (https://www.ncbi.nlm.nih.gov/geo/query/acc.cgi?acc=GSE185547).

## References

[1] Akiyama M, Okada Y, Kanai M, Takahashi A, Momozawa Y, Ikeda M, Iwata N, Ikegawa S, Hirata M, Matsuda K, Iwasaki M, Yamaji T, Sawada N, Hachiya T, Tanno K, Shimizu A, Hozawa A, Minegishi N, Tsugane S (2017). Genome-wide association study identifies 112 new loci for body mass index in the Japanese population. Nat Genet.

[2] Beale EG (2013). Insulin signaling and insulin resistance. J Investig Med.

[3] Bell CG, Walley AJ, Froguel P. The genetics of human obesity. Nat Rev Genet. 2005;6(3). 10.1038/nrg1556. 10.1038/nrg1556 15703762

[4] Buniello A, MacArthur JAL, Cerezo M, Harris LW, Hayhurst J, Malangone C, McMahon A, Morales J, Mountjoy E, Sollis E, Suveges D, Vrousgou O, Whetzel PL, Amode R, Guillen JA, Riat HS, Trevanion SJ, Hall P, Junkins H (2019). The NHGRI-EBI GWAS catalog of published genome-wide association studies, targeted arrays and summary statistics 2019. Nucleic Acids Res.

[5] Chiefari E, Foti DP, Sgarra R, Pegoraro S, Arcidiacono B, Brunetti FS, Greco M, Manfioletti G, Brunetti A (2018). Transcriptional regulation of glucose metabolism: the emerging role of the HMGA1 chromatin factor. Front Endocrinol.

[6] Chiefari E, Nevolo MT, Arcidiacono B, Maurizio E, Nocera A, Iiritano S, Sgarra R, Possidente K, Palmieri C, Paonessa F, Brunetti G, Manfioletti G, Foti D, Brunetti A (2012). HMGA1 is a novel downstream nuclear target of the insulin receptor signaling pathway. Sci Rep.

[7] Churchill GA, Airey DC, Allayee H, Angel JM, Attie AD, Beatty J, et al. The Collaborative Cross, a community resource for the genetic analysis of complex traits. Nat Genet. 2004;36(11). 10.1038/ng1104-1133. 10.1038/ng1104-1133 15514660

[8] Coffey AR, Smallwood TL, Albright J, Hua K, Kanke M, Pomp D, et al. Systems genetics identifies a co-regulated module of liver microRNAs associated with plasma LDL cholesterol in murine diet-induced dyslipidemia. Physiol Genomics. 2017;49(11). 10.1152/physiolgenomics.00050.2017. 10.1152/physiolgenomics.00050.2017 PMC5792136 28916633

[9] Collaborative Cross Consortium. The genome architecture of the Collaborative Cross mouse genetic reference population. Genetics. 2012;190(2). 10.1534/genetics.111.132639. 10.1534/genetics.111.132639 PMC3276630 22345608

[10] Corrêa TAF, Quintanilha BJ, Norde MM, Pinhel MADS, Nonino CB, Rogero MM (2020). Nutritional genomics, inflammation and obesity. Arch Endocrinol Metab.

[11] Creasy SA, Rynders CA, Bergouignan A, Kealey EH, Bessesen DH. Free-living responses in energy balance to short-term overfeeding in adults differing in propensity for obesity. Obesity. 2018;26(4). 10.1002/oby.22121. 10.1002/oby.22121 PMC5868430 29570248

[12] Cuthbertson DJ, Steele T, Wilding JP, Halford JC, Harrold JA, Hamer M, et al. What have human experimental overfeeding studies taught us about adipose tissue expansion and susceptibility to obesity and metabolic complications? Int J Obes. 2017;41(6). 10.1038/ijo.2017.4. 10.1038/ijo.2017.4 28077863

[13] Danforth E. Diet and obesity. Am J Clin Nutr. 1985;41(5). 10.1093/ajcn/41.5.1132. 10.1093/ajcn/41.5.1132 3993620

[14] De Meyts P, Feingold KR, Anawalt B, Boyce A, Chrousos G, de Herder WW, Dhatariya K, Dungan K, Hershman JM, Hofland J, Kalra S, Kaltsas G, Koch C, Kopp P, Korbonits M, Kovacs CS, Kuohung W, Laferrère B, Levy M, McGee EA (2000). The insulin receptor and its signal transduction network. Endotext.

[15] Dejgaard S, Nicolay J, Taheri M, Thomas DY, Bergeron JJM (2004). The ER glycoprotein quality control system. Curr Issues Mol Biol.

[16] Ellero-Simatos S, Fleuren WW, Bauerschmidt S, Dokter WH, Toonen EJ. Identification of gene signatures for prednisolone-induced metabolic dysfunction in collagen-induced arthritic mice. Pharmacogenomics. 2014;15(5). 10.2217/pgs.14.3. 10.2217/pgs.14.3 24798720

[17] Engelking LJ, Kuriyama H, Hammer RE, Horton JD, Brown MS, Goldstein JL, Liang G (2004). Overexpression of Insig-1 in the livers of transgenic mice inhibits SREBP processing and reduces insulin-stimulated lipogenesis. J Clin Invest.

[18] Falconer DS (1989). Introduction to quantitative genetics.

[19] Fernández-Verdejo R, Marlatt KL, Ravussin E, Galgani JE. Contribution of brown adipose tissue to human energy metabolism. Mol Asp Med. 2019;68. 10.1016/j.mam.2019.07.003. 10.1016/j.mam.2019.07.003 PMC7112661 31306668

[20] Festing MFW (1979). Inbred strains in biomedical research.

[21] Fischer IP, Irmler M, Meyer CW, Sachs SJ, Neff F, Hrabě de Angelis M, et al. A history of obesity leaves an inflammatory fingerprint in liver and adipose tissue. Int J Obes. 2017;42(3). 10.1038/ijo.2017.224. 10.1038/ijo.2017.224 PMC5880583 28901330

[22] Fox CS, Liu Y, White CC, Feitosa M, Smith AV, Heard-Costa N, Lohman K, Johnson AD, Foster MC, Greenawalt DM, Griffin P, Ding J, Newman AB, Tylavsky F, Miljkovic I, Kritchevsky SB, GIANT Consortium, MAGIC Consortium, GLGC Consortium (2012). Genome-wide association for abdominal subcutaneous and visceral adipose reveals a novel locus for visceral fat in women. PLoS Genet.

[23] Gautier L, Cope L, Bolstad BM, Irizarry RA. affy—Analysis of Affymetrix GeneChip data at the probe level. Bioinformatics. 2004;20(3). 10.1093/bioinformatics/btg405. 10.1093/bioinformatics/btg405 14960456

[24] Ghazalpour A, Doss S, Zhang B, Wang S, Plaisier C, Castellanos R, Brozell A, Schadt EE, Drake TA, Lusis AJ, Horvath S (2006). Integrating genetic and network analysis to characterize genes related to mouse weight. PLoS Genet.

[25] González-Muniesa P, Mártinez-González M-A, Hu FB, Després J-P, Matsuzawa Y, Loos RJF, et al. Obesity. Nat Rev Dis Primers. 2017;3. 10.1038/nrdp.2017.34. 10.1038/nrdp.2017.34 28617414

[26] Gual P, Baron V, Lequoy V, Van Obberghen E (1998). Interaction of Janus Kinases JAK-1 and JAK-2 with the insulin receptor and the insulin-like growth factor-1 receptor ^1^. Endocrinology.

[27] Haenisch M, Nguyen T, Fihn CA, Goldstein AS, Amory JK, Treuting P, Brabb T, Paik J (2021). Investigation of an ALDH1A1-specific inhibitor for suppression of weight gain in a diet-induced mouse model of obesity. Int J Obes.

[28] Haenisch M, Treuting PM, Brabb T, Goldstein AS, Berkseth K, Amory JK, Paik J (2018). Pharmacological inhibition of ALDH1A enzymes suppresses weight gain in a mouse model of diet-induced obesity. Obes Res Clin Pract.

[29] Hainer V, Zamrazilová H, Spálová J, Hainerová I, Kunesová M, Aldhoon B, Bendlová B (2008). Role of hereditary factors in weight loss and its maintenance. Physiol Res.

[30] Hanke S, Mann M (2009). The phosphotyrosine interactome of the insulin receptor family and its substrates IRS-1 and IRS-2. Mol Cell Proteomics.

[31] Hao L, Huang K-H, Ito K, Sae-tan S, Lambert JD, Ross AC. Fibroblast growth factor 21 (Fgf21) gene expression is elevated in the liver of mice fed a high-carbohydrate liquid diet and attenuated by a lipid emulsion but is not upregulated in the liver of mice fed a high-fat obesogenic diet. J Nutr. 2016;146(2). 10.3945/jn.115.216572. 10.3945/jn.115.216572 PMC4725428 26764334

[32] Hoffmann TJ, Choquet H, Yin J, Banda Y, Kvale MN, Glymour M, Schaefer C, Risch N, Jorgenson E (2018). A large multiethnic genome-wide association study of adult body mass index identifies novel loci. Genetics.

[33] Houtkooper RH, Argmann C, Houten SM, Cantó C, Jeninga EH, Andreux PA, et al. The metabolic footprint of aging in mice. Sci Rep. 2011;1(1). 10.1038/srep00134. 10.1038/srep00134 PMC3216615 22355651

[34] Iglesias-Osma MC, Blanco EJ, Carretero-Hernandez M, Catalano-Iniesta L, Sanchez-Robledo V, Garcia-Barrado MJ, Vicente-Garcia T, Burks DJ, Carretero J (2019). The influence of the lack of insulin receptor substrate 2 (IRS2) on the thyroid gland. Sci Rep.

[35] Iraqi FA, Churchill G, Mott R. The Collaborative Cross, developing a resource for mammalian systems genetics: a status report of the Wellcome Trust cohort. Mamm Genome. 2008;19(6). 10.1007/s00335-008-9113-1. 10.1007/s00335-008-9113-1 18521666

[36] John GK, Mullin GE. The gut microbiome and obesity. Curr Oncol Rep. 2016;18(7). 10.1007/s11912-016-0528-7. 10.1007/s11912-016-0528-7 27255389

[37] Kauffmann A, Gentleman R, Huber W. arrayQualityMetrics—a bioconductor package for quality assessment of microarray data. Bioinformatics. 2008;25(3). 10.1093/bioinformatics/btn647. 10.1093/bioinformatics/btn647 PMC2639074 19106121

[38] Kichaev G, Bhatia G, Loh P-R, Gazal S, Burch K, Freund MK, Schoech A, Pasaniuc B, Price AL (2019). Leveraging polygenic functional enrichment to improve GWAS power. Am J Hum Genet.

[39] Kiefer FW, Orasanu G, Nallamshetty S, Brown JD, Wang H, Luger P, Qi NR, Burant CF, Duester G, Plutzky J (2012). Retinaldehyde dehydrogenase 1 coordinates hepatic gluconeogenesis and lipid metabolism. Endocrinology.

[40] Kim O-K, Jun W, Lee J (2015). Mechanism of ER stress and inflammation for hepatic insulin resistance in obesity. Ann Nutr Metab.

[41] Kozul CD, Nomikos AP, Hampton TH, Warnke LA, Gosse JA, Davey JC, et al. Laboratory diet profoundly alters gene expression and confounds genomic analysis in mouse liver and lung. Chem Biol Interact. 2008;173(2). 10.1016/j.cbi.2008.02.008. 10.1016/j.cbi.2008.02.008 18396267

[42] Kushi R, Hirota Y, Ogawa W (2021). Insulin resistance and exaggerated insulin sensitivity triggered by single-gene mutations in the insulin signaling pathway. Diabetol Int.

[43] Kwok A, Zvetkova I, Virtue S, Luijten I, Huang-Doran I, Tomlinson P, Bulger DA, West J, Murfitt S, Griffin J, Alam R, Hart D, Knox R, Voshol P, Vidal-Puig A, Jensen J, O’Rahilly S, Semple RK (2020). Truncation of Pik3r1 causes severe insulin resistance uncoupled from obesity and dyslipidaemia by increased energy expenditure. Mol Metab.

[44] Landrier J-F, Kasiri E, Karkeni E, Mihály J, Béke G, Weiss K, Lucas R, Aydemir G, Salles J, Walrand S, de Lera AR, Rühl R (2017). Reduced adiponectin expression after high-fat diet is associated with selective up-regulation of ALDH1A1 and further retinoic acid receptor signaling in adipose tissue. FASEB J.

[45] Langfelder P, Horvath S. WGCNA: an R package for weighted correlation network analysis. BMC Bioinformatics. 2008;9(1). 10.1186/1471-2105-9-559. 10.1186/1471-2105-9-559 PMC2631488 19114008

[46] Langhans W. Role of the liver in the control of glucose-lipid utilization and body weight. Curr Opin Clin Nutr Metab Care. 2003;6(4). 10.1097/01.mco.0000078993.96795.16. 10.1097/01.mco.0000078993.96795.16 12806220

[47] Lightfoot JT, Turner MJ, Debate KA, Kleeberger SR. Interstrain variation in murine aerobic capacity. Med Sci Sports Exerc. 2001;33(12). 10.1097/00005768-200112000-00012. 10.1097/00005768-200112000-00012 11740298

[48] Lin X, Taguchi A, Park S, Kushner JA, Li F, Li Y, White MF (2004). Dysregulation of insulin receptor substrate 2 in beta cells and brain causes obesity and diabetes. J Clin Invest.

[49] Lindgren CM, Heid IM, Randall JC, Lamina C, Steinthorsdottir V, Qi L, Speliotes EK, Thorleifsson G, Willer CJ, Herrera BM, Jackson AU, Lim N, Scheet P, Soranzo N, Amin N, Aulchenko YS, Chambers JC, Drong A, Luan J (2009). Genome-wide association scan meta-analysis identifies three loci influencing adiposity and fat distribution. PLoS Genet.

[50] Lis JT. A 50 year history of technologies that drove discovery in eukaryotic transcription regulation. Nat Struct Mol Biol. 2019;26(9). 10.1038/s41594-019-0288-9. 10.1038/s41594-019-0288-9 PMC7106917 31439942

[51] Locke AE, Kahali B, Berndt SI, Justice AE, Pers TH, Day FR, Powell C, Vedantam S, Buchkovich ML, Yang J, Croteau-Chonka DC, Esko T, Fall T, Ferreira T, Gustafsson S, Kutalik Z, Luan J, Mägi R, Randall JC (2015). Genetic studies of body mass index yield new insights for obesity biology. Nature.

[52] Loos RJ. The genetics of adiposity. Curr Opin Genet Dev. 2018;50. 10.1016/j.gde.2018.02.009. 10.1016/j.gde.2018.02.009 PMC6089650 29529423

[53] Ludwig DS, Aronne LJ, Astrup A, de Cabo R, Cantley LC, Friedman MI, et al. The carbohydrate-insulin model: a physiological perspective on the obesity pandemic. Am J Clin Nutr. 2021;nqab270. 10.1093/ajcn/nqab270. 10.1093/ajcn/nqab270 PMC8634575 34515299

[54] Luke SG. Evaluating significance in linear mixed-effects models in R. Behav Res Methods. 2016;49(4). 10.3758/s13428-016-0809-y. 10.3758/s13428-016-0809-y 27620283

[55] Luo G, Hurtig M, Zhang X, Nilsson-Ehle P, Xu N (2005). Leptin inhibits apolipoprotein M transcription and secretion in human hepatoma cell line, HepG2 cells. Biochim Biophys Acta.

[56] Luo Y, Burrington CM, Graff EC, Zhang J, Judd RL, Suksaranjit P, et al. Metabolic phenotype and adipose and liver features in a high-fat Western diet-induced mouse model of obesity-linked NAFLD. Am J Physiol Endocrinol Metab. 2016;310(6). 10.1152/ajpendo.00319.2015. 10.1152/ajpendo.00319.2015 PMC4796265 26670487

[57] Martinez KB, Pierre JF, Chang EB. The gut microbiota. Gastroenterol Clin N Am. 2016;45(4). 10.1016/j.gtc.2016.07.001. 10.1016/j.gtc.2016.07.001 PMC5127273 27837775

[58] Miao J, Ling AV, Manthena PV, Gearing ME, Graham MJ, Crooke RM, Croce KJ, Esquejo RM, Clish CB, Vicent D, Biddinger SB, Morbid Obesity Study Group (2015). Flavin-containing monooxygenase 3 as a potential player in diabetes-associated atherosclerosis. Nat Commun.

[59] Moisan A, Lee Y-K, Zhang JD, Hudak CS, Meyer CA, Prummer M, Zoffmann S, Truong HH, Ebeling M, Kiialainen A, Gérard R, Xia F, Schinzel RT, Amrein KE, Cowan CA (2015). White-to-brown metabolic conversion of human adipocytes by JAK inhibition. Nat Cell Biol.

[60] Nicoletti CF, Azevedo RG, Pinhel MAS, Delfino HBP, Nonino CB. INSIG2 gene polymorphism is associated with higher blood pressure and triglyceride levels in Brazilian obese subjects. Nutr Hosp. 2019. 10.20960/nh.2359. 10.20960/nh.2359 31144980

[61] Ouyang S, Mo Z, Sun S, Yin K, Lv Y (2020). Emerging role of Insig-1 in lipid metabolism and lipid disorders. Clin Chim Acta.

[62] Ozcan U (2004). Endoplasmic reticulum stress links obesity, insulin action, and type 2 diabetes. Science.

[63] Parida S, Siddharth S, Sharma D (2019). Adiponectin, obesity, and cancer: clash of the bigwigs in health and disease. Int J Mol Sci.

[64] Philip VM, Sokoloff G, Ackert-Bicknell CL, Striz M, Branstetter L, Beckmann MA, et al. Genetic analysis in the Collaborative Cross breeding population. Genome Res. 2011;21(8). 10.1101/gr.113886.110. 10.1101/gr.113886.110 PMC3149490 21734011

[65] Phillips IR, Shephard EA (2020). Flavin-containing monooxygenase 3 (FMO3): genetic variants and their consequences for drug metabolism and disease. Xenobiotica.

[66] Phipson B, Lee S, Majewski IJ, Alexander WS, Smyth GK. Robust hyperparameter estimation protects against hypervariable genes and improves power to detect differential expression. Ann Appl Stat. 2016;10(2). 10.1214/16-aoas920. 10.1214/16-AOAS920 PMC5373812 28367255

[67] Plourde M, Vohl M-C, Bellis C, Carless M, Dyer T, Dolley G, Marette A, Després J-P, Bouchard C, Blangero J, Pérusse L (2013). A variant in the LRRFIP1 gene is associated with adiposity and inflammation: a variant in the LRRFIP1 gene. Obesity.

[68] Prakash J, Mittal B, Apurva S, Shally A, Pranjal S, Neena S (2017). Common genetic variant of insig2 gene rs7566605 polymorphism is associated with severe obesity in North India. Iran Biomed J.

[69] Pulit SL, Stoneman C, Morris AP, Wood AR, Glastonbury CA, Tyrrell J, Yengo L, Ferreira T, Marouli E, Ji Y, Yang J, Jones S, Beaumont R, Croteau-Chonka DC, Winkler TW, Hattersley AT, Loos RJF, Hirschhorn JN, GIANT Consortium (2019). Meta-analysis of genome-wide association studies for body fat distribution in 694 649 individuals of European ancestry. Hum Mol Genet.

[70] Que E, James KL, Coffey AR, Smallwood TL, Albright J, Huda MN, et al. Genetic architecture modulates diet-induced hepatic mRNA and miRNA expression profiles in diversity outbred mice. Genetics. 2020;216(1). 10.1534/genetics.120.303481. 10.1534/genetics.120.303481 PMC7463293 32763908

[71] R Core Team (2019). R: a language and environment for statistical computing.

[72] Rask-Andersen M, Karlsson T, Ek WE, Johansson Å (2019). Genome-wide association study of body fat distribution identifies adiposity loci and sex-specific genetic effects. Nat Commun.

[73] Ritchie ME, Phipson B, Wu D, Hu Y, Law CW, Shi W, et al. Limma powers differential expression analyses for RNA-sequencing and microarray studies. Nucleic Acids Res. 2015;43(7). 10.1093/nar/gkv007. 10.1093/nar/gkv007 PMC4402510 25605792

[74] Roeder RG. 50+ years of eukaryotic transcription: an expanding universe of factors and mechanisms. Nat Struct Mol Biol. 2019;26(9). 10.1038/s41594-019-0287-x. 10.1038/s41594-019-0287-x PMC6867066 31439941

[75] Rui L, Terjung R (2014). Energy metabolism in the liver. Comprehensive physiology.

[76] Schmidt SL, Harmon KA, Sharp TA, Kealey EH, Bessesen DH. The Effects of overfeeding on spontaneous physical activity in obesity prone and obesity resistant humans. Obesity. 2012;20(11). 10.1038/oby.2012.103. 10.1038/oby.2012.103 PMC3782097 22522883

[77] Shannon P, Markiel A, Ozier O, Baliga NS, Wang JT, Ramage D, Amin N, Schwikowski B, Ideker T (2003). Cytoscape: a software environment for integrated models of biomolecular interaction networks. Genome Res.

[78] Shih DM, Wang Z, Lee R, Meng Y, Che N, Charugundla S, Qi H, Wu J, Pan C, Brown JM, Vallim T, Bennett BJ, Graham M, Hazen SL, Lusis AJ (2015). Flavin containing monooxygenase 3 exerts broad effects on glucose and lipid metabolism and atherosclerosis. J Lipid Res.

[79] Shorter JR, Najarian ML, Bell TA, Blanchard M, Ferris MT, Hock P, et al. Whole genome sequencing and progress toward full inbreeding of the mouse collaborative cross population. G3. 2019;9(5). 10.1534/g3.119.400039. 10.1534/g3.119.400039 PMC6505143 30858237

[80] Silva JP, van Booven D. Analysis of diet-induced differential methylation, expression, and interactions of lncRNA and protein-coding genes in mouse liver. Sci Rep. 2018;8(1). 10.1038/s41598-018-29993-4. 10.1038/s41598-018-29993-4 PMC6070528 30069000

[81] Sims EAH. Experimental obesity, dietary-induced thermogenesis, and their clinical implications. Clin Endocrinol Metab. 1976;5(2). 10.1016/s0300-595x(76)80027-8. 10.1016/s0300-595x(76)80027-8 782745

[82] Singh RK, Kumar P, Mahalingam K. Molecular genetics of human obesity: a comprehensive review. C R Biol. 2017;340(2). 10.1016/j.crvi.2016.11.007. 10.1016/j.crvi.2016.11.007 28089486

[83] Smith EM, Zhang Y, Baye TM, Gawrieh S, Cole R, Blangero J, Carless MA, Curran JE, Dyer TD, Abraham LJ, Moses EK, Kissebah AH, Martin LJ, Olivier M (2010). INSIG1 influences obesity-related hypertriglyceridemia in humans. J Lipid Res.

[84] Speakman JR (2018). Obesity and thermoregulation. Handb Clin Neurol.

[85] Srivastava A, Morgan AP, Najarian ML, Sarsani VK, Sigmon JS, Shorter JR, et al. Genomes of the mouse Collaborative Cross. Genetics. 2017;206(2). 10.1534/genetics.116.198838. 10.1534/genetics.116.198838 PMC5499171 28592495

[86] Swanzey E, O’Connor C, Reinholdt LG (2021). Mouse genetic reference populations: cellular platforms for integrative systems genetics. Trends Genet.

[87] Tachmazidou I, Süveges D, Min JL, Ritchie GRS, Steinberg J, Walter K, Iotchkova V, Schwartzentruber J, Huang J, Memari Y, McCarthy S, Crawford AA, Bombieri C, Cocca M, Farmaki A-E, Gaunt TR, Jousilahti P, Kooijman MN, Lehne B (2017). Whole-genome sequencing coupled to imputation discovers genetic signals for anthropometric traits. Am J Hum Genet.

[88] Locke AE, Kahali B, Berndt SI, Justice AE, Pers TH, The LifeLines Cohort Study, The ADIPOGen Consortium, The AGEN-BMI Working Group, The CARDIOGRAMplusC4D Consortium, The CKDGen Consortium, The GLGC, The ICBP, The MAGIC Investigators, The MuTHER Consortium, The MIGen Consortium, The PAGE Consortium, The ReproGen Consortium, The GENIE Consortium, The International Endogene Consortium (2015). Genetic studies of body mass index yield new insights for obesity biology. Nature.

[89] Threadgill DW, Churchill GA. Ten years of the Collaborative Cross. Genetics. 2012;190(2). 10.1534/genetics.111.138032. 10.1534/genetics.111.138032 PMC3276648 22345604

[90] Timper K, Brüning JC. Hypothalamic circuits regulating appetite and energy homeostasis: pathways to obesity. Dis Model Mech. 2017;10(6). 10.1242/dmm.026609. 10.1242/dmm.026609 PMC5483000 28592656

[91] Torres-Fuentes C, Schellekens H, Dinan TG, Cryan JF. The microbiota–gut–brain axis in obesity. Lancet Gastroenterol Hepatol. 2017;2(10). 10.1016/s2468-1253(17)30147-4. 10.1016/S2468-1253(17)30147-4 28844808

[92] Treacy E (1998). Mutations of the flavin-containing monooxygenase gene (FMO3) cause trimethylaminuria, a defect in detoxication. Hum Mol Genet.

[93] Trefts E, Gannon M, Wasserman DH. The liver. Curr Biol. 2017;27(21). 10.1016/j.cub.2017.09.019. 10.1016/j.cub.2017.09.019 PMC5897118 29112863

[94] Wang H, Zhang F, Zeng J, Wu Y, Kemper KE, Xue A, Zhang M, Powell JE, Goddard ME, Wray NR, Visscher PM, McRae AF, Yang J (2019). Genotype-by-environment interactions inferred from genetic effects on phenotypic variability in the UK Biobank. Sci Adv.

[95] Wang Z, Levison BS, Hazen JE, Donahue L, Li X-M, Hazen SL. Measurement of trimethylamine-N-oxide by stable isotope dilution liquid chromatography tandem mass spectrometry. Anal Biochem. 2014;455. 10.1016/j.ab.2014.03.016. 10.1016/j.ab.2014.03.016 PMC4167037 24704102

[96] Warrier M, Shih DM, Burrows AC, Ferguson D, Gromovsky AD, Brown AL, Marshall S, McDaniel A, Schugar RC, Wang Z, Sacks J, Rong X, Vallim TDA, Chou J, Ivanova PT, Myers DS, Brown HA, Lee RG, Crooke RM (2015). The TMAO-generating enzyme flavin monooxygenase 3 is a central regulator of cholesterol balance. Cell Rep.

[97] Williams EP, Mesidor M, Winters K, Dubbert PM, Wyatt SB. Overweight and obesity: prevalence, consequences, and causes of a growing public health problem. Curr Obes Rep. 2015;4(3). 10.1007/s13679-015-0169-4. 10.1007/s13679-015-0169-4 26627494

[98] Winkler TW, Justice AE, Graff M, Barata L, Feitosa MF, Chu S, Czajkowski J, Esko T, Fall T, Kilpeläinen TO, Lu Y, Mägi R, Mihailov E, Pers TH, Rüeger S, Teumer A, Ehret GB, Ferreira T, Heard-Costa NL (2015). The influence of age and sex on genetic associations with adult body size and shape: a large-scale genome-wide interaction study. PLoS Genet.

[99] Xu N, Dahlbäck B (1999). A novel human apolipoprotein (apoM). J Biol Chem.

[100] Yabe D, Brown MS, Goldstein JL (2002). Insig-2, a second endoplasmic reticulum protein that binds SCAP and blocks export of sterol regulatory element-binding proteins. Proc Natl Acad Sci.

[101] Yam P, Albright J, VerHague M, Gertz ER, Pardo-Manuel de Villena F, Bennett BJ (2021). Genetic background shapes phenotypic response to diet for adiposity in the collaborative cross. Front Genet.

[102] Yamaguchi A, Hori O, Stern DM, Hartmann E, Ogawa S, Tohyama M (1999). Stress-associated endoplasmic reticulum protein 1 (Serp1)/ribosome-associated membrane protein 4 (Ramp4) stabilizes membrane proteins during stress and facilitates subsequent glycosylation. J Cell Biol.

[103] Yamauchi T, Kamon J, Ito Y, Tsuchida A, Yokomizo T, Kita S, Sugiyama T, Miyagishi M, Hara K, Tsunoda M, Murakami K, Ohteki T, Uchida S, Takekawa S, Waki H, Tsuno NH, Shibata Y, Terauchi Y, Froguel P (2003). Cloning of adiponectin receptors that mediate antidiabetic metabolic effects. Nature.

[104] Yang T, Espenshade PJ, Wright ME, Yabe D, Gong Y, Aebersold R, Goldstein JL, Brown MS (2002). Crucial step in cholesterol homeostasis. Cell.

[105] Yilmaz E, Engin AB, Engin A (2017). Endoplasmic reticulum stress and obesity. Obesity and lipotoxicity.

[106] Zhang B, Horvath S. A general framework for weighted gene co-expression network analysis. Stat Appl Genet Mol Biol. 2005;4(1). 10.2202/1544-6115.1128. 10.2202/1544-6115.1128 16646834

[107] Zhao W, Langfelder P, Fuller T, Dong J, Li A, Hovarth S (2010). Weighted gene coexpression network analysis: state of the art. J Biopharm Stat.

[108] Zhu Z, Guo Y, Shi H, Liu C-L, Panganiban RA, Chung W, O’Connor LJ, Himes BE, Gazal S, Hasegawa K, Camargo CA, Qi L, Moffatt MF, Hu FB, Lu Q, Cookson WOC, Liang L (2020). Shared genetic and experimental links between obesity-related traits and asthma subtypes in UK Biobank. J Allergy Clin Immunol.

