# Supplementary material for: Altered macronutrient composition and genetics influence the complex transcriptional network associated with adiposity in the Collaborative Cross
Source: Genes Nutr. 2022 Aug 10;17:13. doi: 10.1186/s12263-022-00714-x (PMC9364539; doi:10.1186/s12263-022-00714-x)

**Supplementary Figure 1. Analyses framework and main results**

Figure depicts the analyses pipelines using data from 22 CC strains (n = 123) centered around (A) Linear Models for MicroArray (limma) to examine hepatic gene expression at the individual gene level while also incorporating correlation analysis with phenotypic data and utilizing the GWAS catalog to guide the search for clinically relevant genes in the study, and (B) Weighted gene co-expression network analysis (WGCNA) to investigate expression of groups of related genes, which also integrates correlation analysis between module eigengenes (estimations of related groups of genes) and phenotypic data. Analyses performed in each pipeline are indicated by italics and data used for each analysis are connected by lines. For example, microarray and phenotype data were used together (indicated by the bracket connecting the “microarray data” and “phenotype data” boxes) to perform phenotype x gene expression correlations (indicated by the text in italics), which generated a list of genes whose expression levels were significantly correlated with body fat % (BF%) (represented by the blue box under the arrow).

**
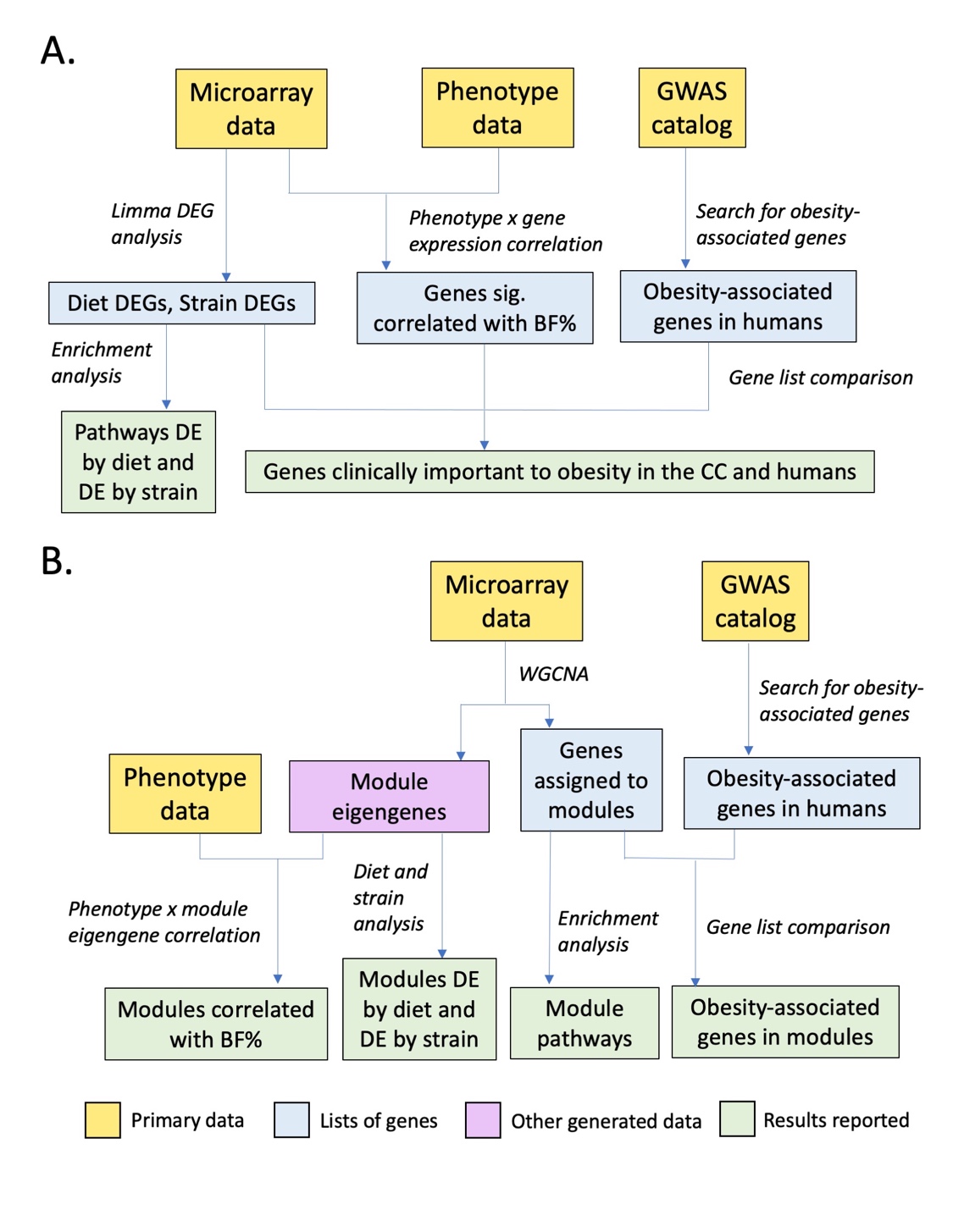
Supplementary Figure 2. Dendrogram WGCNA identifies gene co-regulated modules**

Using the cleaned and filtered hepatic gene expression data from mice fed the HP diet and mice fed the HS diet, WGCNA identified 13 modules with arbitrarily assigned colors. The 11,542 expressed genes from the limma analysis were used to form the modules, which varied widely in terms of the number genes within each module. Dendrogram was created using the *blockwiseModules* function to construct the unsigned network in one block, calculate an adjacency matrix with Pearson correlations, calculate the topological overlap matrix (TOM) using the signed method, cluster genes using the default average linkage hierarchical clustering, and establish modules by the Dynamic Hybrid tree cut method.

**Supplementary Figure 3. Connectivity for genes assigned to the magenta module**

A visual representation of the network structure for genes within a module using the magenta module as an example. Each node represents a gene assigned to the magenta module, where edges depict the number of connections between genes.

**
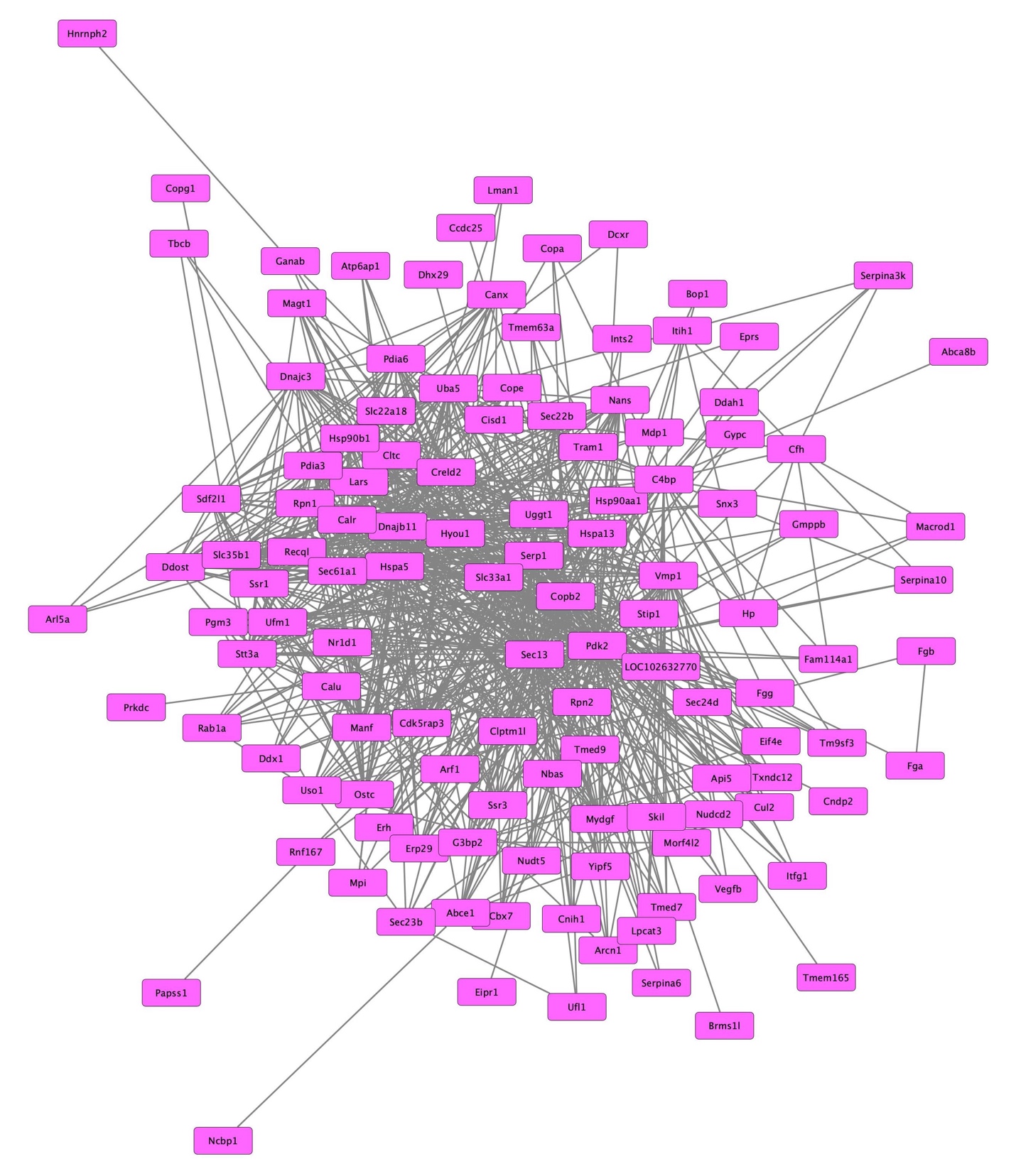
**

**Supplementary Figure 4. Significantly enriched pathways and ontologies for the magenta module**

Genes in the magenta module were significantly enriched in 35 GO Biological Processes, four GO Molecular Functions, two Jensen Diseases, and two KEGG pathways in mice related to endoplasmic reticulum function. The top 10 most significantly enriched GO Biological Processes are shown. The number of genes present in the magenta module and total number of genes that belong to respective enriched pathways/ontologies are displayed in each bar. All significant enrichment terms are shown in Supplementary Table 11, Additional File 2.


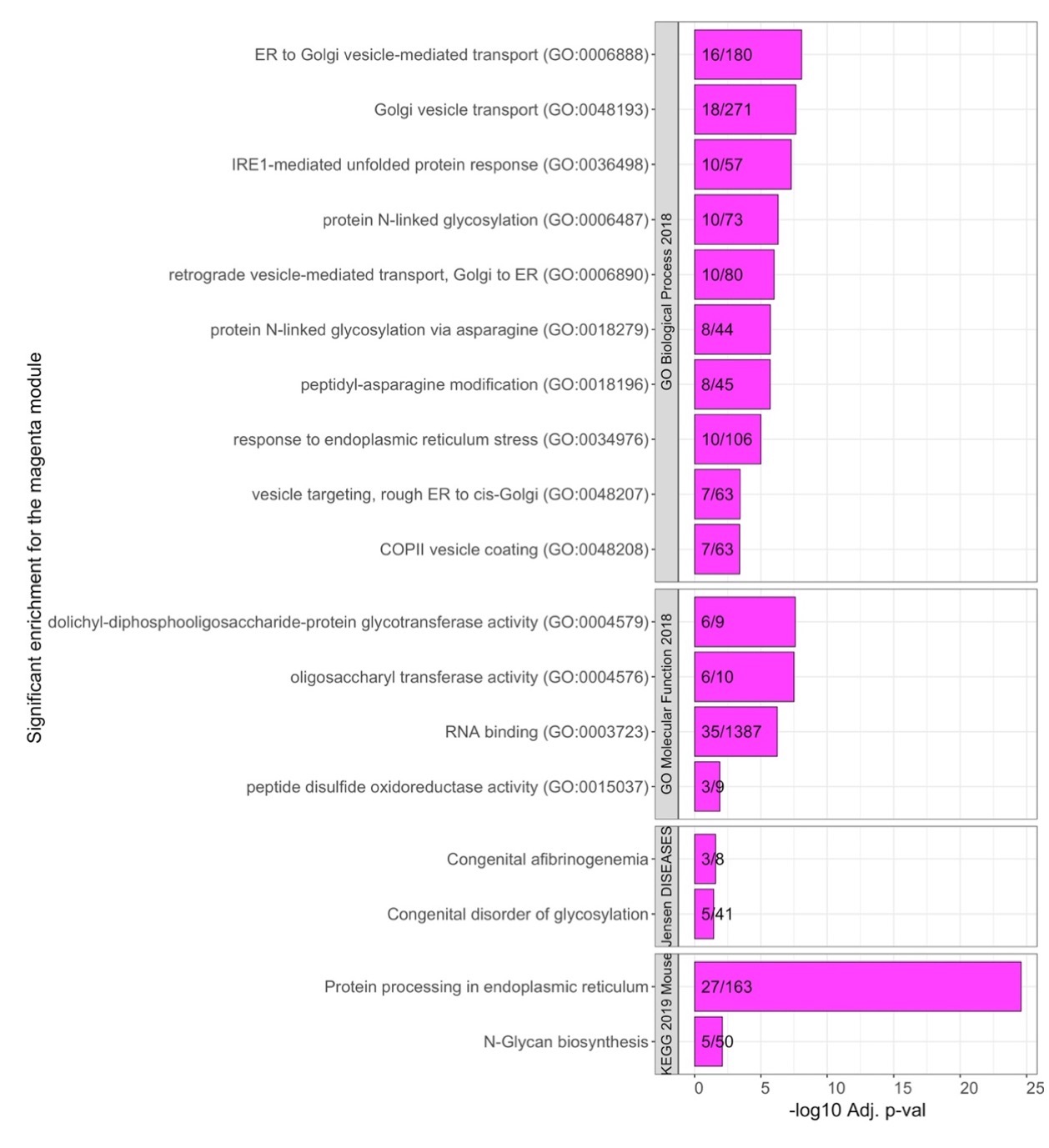


**Supplementary Figure 5. Significantly enriched pathways and ontologies for the red module**

Genes in the red module were significantly enriched in 37 GO Biological Processes, two GO Molecular Functions (MF), three Jensen Diseases, and 24 KEGG pathways in mice related to steroid, cholesterol, and fatty acid biosynthesis/metabolism. The top 10 most significantly enriched GO Biological Processes and KEGG pathways are shown. The number of genes present in the red module and total number of genes that belong to respective enriched pathways/ontologies are displayed in each bar. All significant enrichment terms are shown in Supplementary Table 11, Additional File 2.


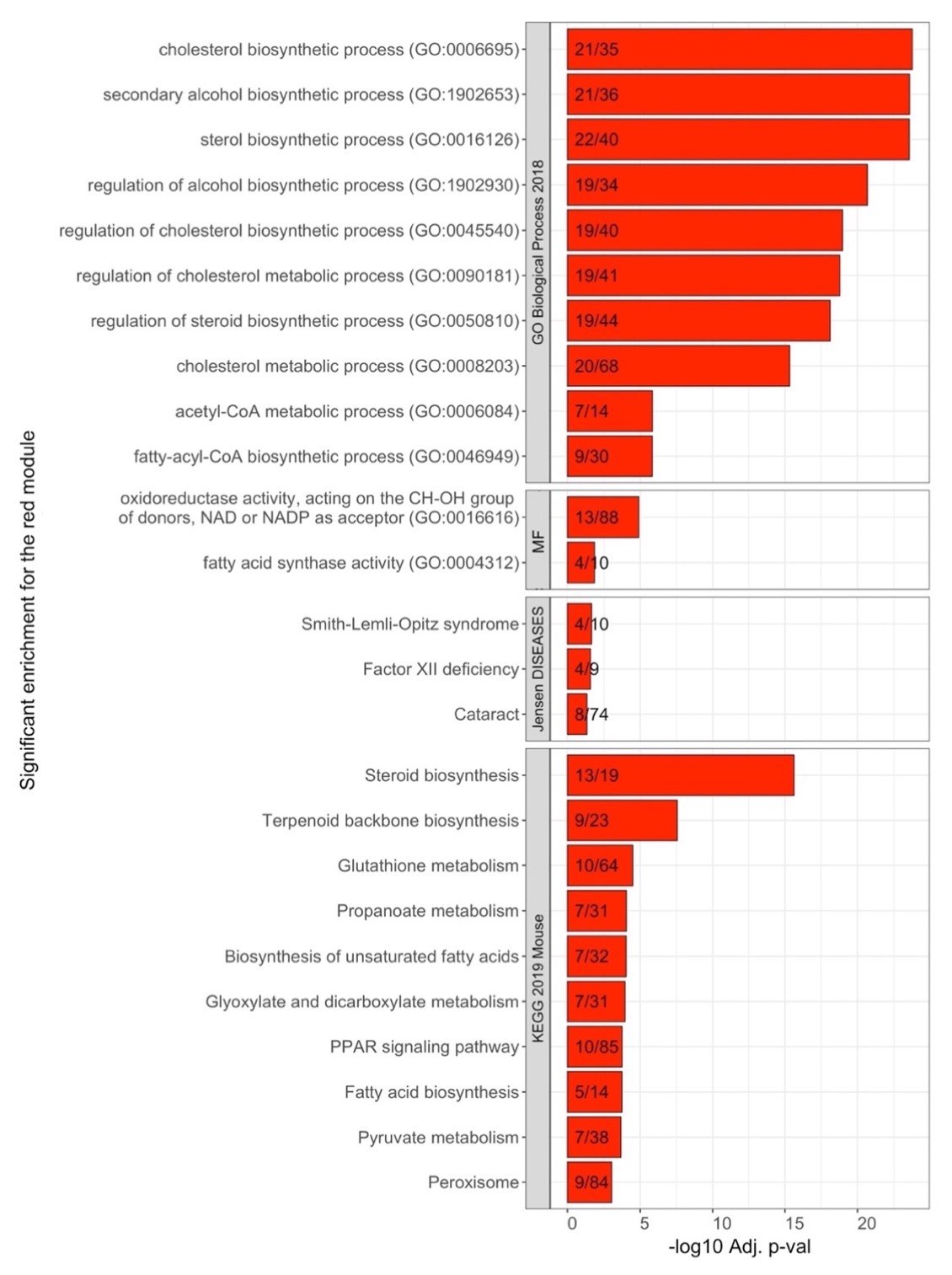


**Supplementary Figure 6. Significantly enriched pathways and ontologies for the yellow module**

Genes in the yellow module were significantly enriched in 27 GO Biological Processes, 11 GO Molecular Functions, two Jensen Diseases (J’s DISEASES), and six KEGG pathways in mice related to a variety of functions such as photoperiodism, transcription regulation, insulin signaling, etc. The top 10 most significantly enriched GO Biological Processes and Molecular Functions are shown. The number of genes present in the yellow module and total number of genes that belong to respective enriched pathways/ontologies are displayed in each bar. All significant enrichment terms are shown in Supplementary Table 11, Additional File 2.


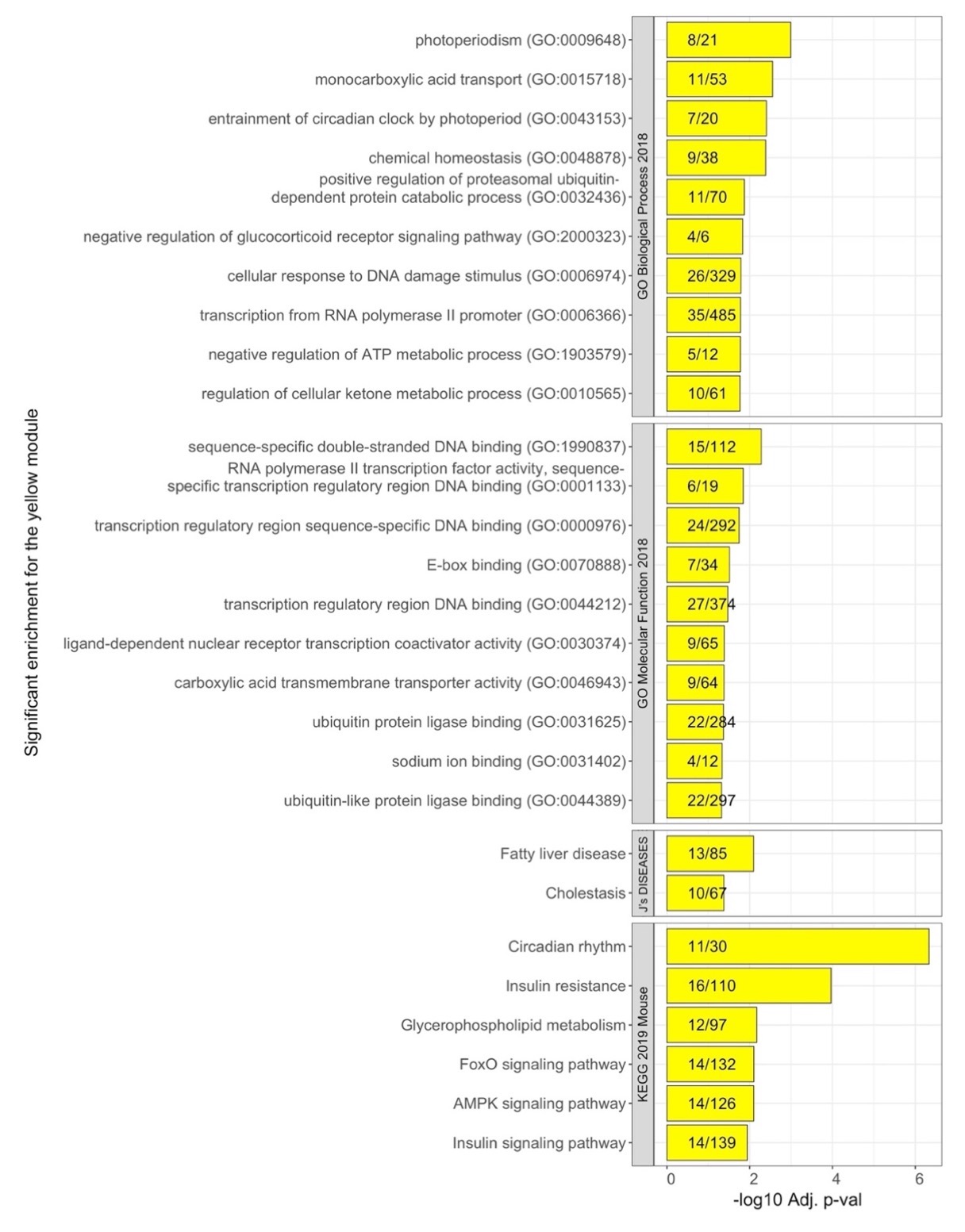


**Supplementary Figure 7. Correlations between the magenta, red, and yellow MEs with body fat % by diet**

Spearman’s correlations performed between the magenta, red, and yellow module eigengenes (PC1) and body fat % by diet suggest that the magnitude and direction of correlation in gene expression between these modules change depending on diet.


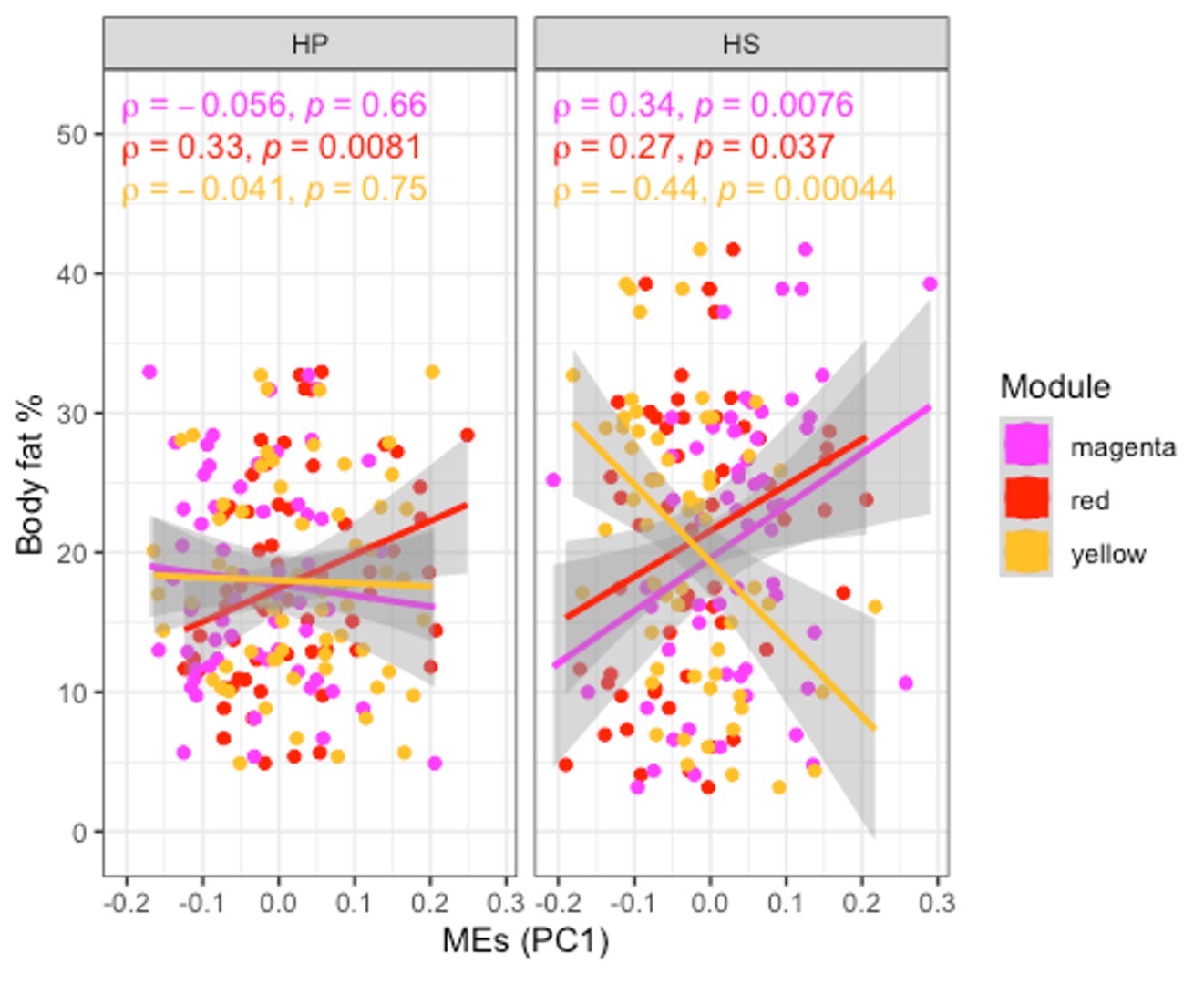

Supplement: Supplementary file 1 — Additional file 1: Supplementary Figure 1. Analyses framework and main results. Supplementary Figure 2. Dendrogram WGCNA identifies gene co-regulated modules. Supplementary Figure 3. Connectivity for genes assigned to the magenta module. Supplementary Figure 4. Significantly enriched pathways and ontologies for the magenta module. Supplementary Figure 5. Significantly enriched pathways and ontologies for the red module. Supplementary Figure 6. Significantly enriched pathways and ontologies for the yellow module. Supplementary Figure 7. Correlations between the magenta, red, and yellow MEs with body fat % by diet. [file 12263_2022_714_MOESM1_ESM.docx]
